# Supplementary material for: Silver Catalyzed Decarbonylative [3 + 2] Cycloaddition of Cyclobutenediones and Formamides
Source: Molecules. 2021 May 17;26(10):2974. doi: 10.3390/molecules26102974 (PMC8156422; doi:10.3390/molecules26102974)
Supplement: Supplementary file 1 [file molecules-26-02974-s001.zip › molecules-1202617-supplementary.pdf]

Supporting Information

Silver Catalyzed Decarbonylative [3 + 2] Cycloaddition of Cyclobutenediones and Formamides

Pengcheng Wang[+], Ruirui Yu[+], Sajjad Ali, Zhengshen Wang[*], Zhigang Liu, Jin-Ming Gao, Huaiji Zheng[*]

Shaanxi Key Laboratory of Natural Products and Chemical Biology, College of Chemistry and Pharmacy, Northwest Agriculture and Forestry University, 3 Taicheng Road, Yangling 712100, China

wangzs@nwsuaf.edu.cn (Z.W.); hjzheng@nwsuaf.edu.cn (H.Z.)

Table of Contents

1. X-ray Structure of **3aa**	S2
2. ^{1}H and ^{13}C NMR spectra data	S10

1. X-ray Structure of 3aa (CCDC: **2036646**)

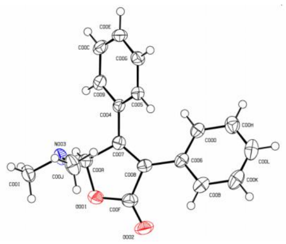

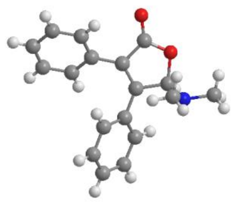

Table 1. Sample and crystal data for **3aa.**

Identification code	**3aa**	
Chemical formula	$C_{18}H_{17}NO_2$	
Formula weight	279.32 g/mol	
Temperature	150(2) K	
Wavelength	1.34139 Å	
Crystal size	0.200 x 0.200 x 0.200 mm	
Crystal habit	colorless block	
Crystal system	orthorhombic	
Space group	P 21 21 21	
	a = 8.6663(15) Å	α = 90°
Unit cell dimensions	b = 10.2215(16) Å	β = 90°
	c = 16.751(3) Å	γ = 90°
Volume	1483.8(4) $Å^3$	
Z	4	
Density (calculated)	1.250 g/cm^3	
Absorption coefficient	0.414 mm^{-1}	
F(000)	592	

Table 2. Data collection and structure refinement for **3aa.**

Theta range for data collection	4.59 to 53.90 °
Index ranges	-9<=h<=10,-11<=k<=12,-18<=l<=20
Reflections collected	13576
Independent reflections	2582 [R(int) = 0.0345]
Coverage of independent reflections	96.9%
Absorption correction	Multi-Scan
Structure solution technique	direct methods
Structure solution program	SHELXT 2014/5 (Sheldrick, 2014)
Refinement method	Full-matrix least-squares on F^2
Refinement program	SHELXL-2018/3 (Sheldrick, 2018)
Function minimized	Σ w(F_o^2 - F_c^2)^2
Data / restraints / parameters	2582 / 0 / 192
Goodness-of-fit on F^2	1.080
Final R indices	2549data;I>2σ(I) R1=0.0277,wR2=0.0774
	all data R1=0.0281,wR2=0.0778
Weighting scheme	w=1/[σ^2(F_o^2)+(0.0443P)^2+0.1633P]
	where P=(F_o^2+2F_c^2)/3
Absolute structure parameter	0.5
Largest diff. peak and hole	0.136 and -0.117 eÅ^{-3}
R.M.S. deviation from mean	0.028 eÅ^{-3}

Table 3. Atomic coordinates and equivalent isotropic atomic displacement parameters

(Å^2) for **3aa**.

U(eq) is defined as one third of the trace of the orthogonalized U_{ij} tensor.

	x/a	y/b	z/c	U(eq)
O001	0.12176(16)	0.20329(11)	0.73545(7)	0.0397(3)
O002	0.11563(18)	0.03077(12)	0.65347(9)	0.0488(4)
N003	0.28030(17)	0.38069(15)	0.78404(8)	0.0355(4)
C00A	0.1626(2)	0.34401(16)	0.73009(10)	0.0315(4)
C00B	0.28372(19)	0.09394(17)	0.49742(11)	0.0375(4)
C00C	0.2381(2)	0.72415(17)	0.60825(10)	0.0376(4)
C00D	0.1828(2)	0.30379(17)	0.45939(10)	0.0337(4)
C00E	0.3672(2)	0.73008(16)	0.55968(10)	0.0373(4)
C00F	0.1405(2)	0.14616(16)	0.66337(11)	0.0360(4)
C00G	0.4464(2)	0.61696(17)	0.54032(10)	0.0335(4)
C00H	0.2112(2)	0.2756(2)	0.37980(10)	0.0404(4)
C00I	0.2338(3)	0.36610(19)	0.86754(10)	0.0434(5)
C00J	0.4301(2)	0.3221(2)	0.76861(11)	0.0484(5)
C00K	0.3103(2)	0.0651(2)	0.41726(12)	0.0450(5)
C00L	0.2745(2)	0.1557(2)	0.35877(11)	0.0451(5)
C004	0.26265(19)	0.48847(15)	0.61489(9)	0.0263(3)
C005	0.39376(19)	0.49645(15)	0.56697(9)	0.0289(4)
C006	0.21979(18)	0.21437(16)	0.51949(10)	0.0302(3)
C007	0.20701(18)	0.36063(15)	0.64331(9)	0.0276(3)

C008	0.19310(19)	0.24610(15)	0.60447(10)	0.0296(4)
C009	0.1868(2)	0.60431(16)	0.63650(10)	0.0324(4)

Table 4. Bond lengths (Å) for **3aa**.

O001-C00F	1.351(2)	O001-C00A	1.484(2)
O002-C00F	1.210(2)	N003-C00A	1.413(2)
N003-C00J	1.453(2)	N003-C00I	1.463(2)
C00A-C007	1.513(2)	C00A-H00A	1.0
C00B-C00K	1.394(3)	C00B-C006	1.400(2)
C00B-H00B	0.95	C00C-C00E	1.385(3)
C00C-C009	1.386(2)	C00C-H00C	0.95
C00D-C00H	1.386(2)	C00D-C006	1.397(2)
C00D-H00D	0.95	C00E-C00G	1.383(3)
C00E-H00E	0.95	C00F-C008	1.492(2)
C00G-C005	1.387(2)	C00G-H00G	0.95
C00H-C00L	1.387(3)	C00H-H00H	0.95
C00I-H00F	0.98	C00I-H00I	0.98
C00I-H00J	0.98	C00J-H00K	0.98
C00J-H00L	0.98	C00J-H00M	0.98
C00K-C00L	1.383(3)	C00K-H00N	0.95
C00L-H00O	0.95	C004-C005	1.394(2)
C004-C009	1.402(2)	C004-C007	1.472(2)
C005-H005	0.95	C006-C008	1.478(2)

C007-C008	1.345(2)	C009-H009	0.95

Table 5. Bond angles (°) for **3aa**.

C00F-O001-C00A	109.65(13)	C00A-N003-C00J	114.94(14)
C00A-N003-C00I	112.68(15)	C00J-N003-C00I	111.96(15)
N003-C00A-O001	112.99(14)	N003-C00A-C007	113.63(14)
O001-C00A-C007	103.15(13)	N003-C00A-H00A	108.9
O001-C00A-H00A	108.9	C007-C00A-H00A	108.9
C00K-C00B-C006	120.37(18)	C00K-C00B-H00B	119.8
C006-C00B-H00B	119.8	C00E-C00C-C009	119.88(15)
C00E-C00C-H00C	120.1	C009-C00C-H00C	120.1
C00H-C00D-C006	121.09(16)	C00H-C00D-H00D	119.5
C006-C00D-H00D	119.5	C00G-C00E-C00C	120.15(16)
C00G-C00E-H00E	119.9	C00C-C00E-H00E	119.9
O002-C00F-O001	121.48(16)	O002-C00F-C008	129.13(17)
O001-C00F-C008	109.39(14)	C00E-C00G-C005	120.23(16)
C00E-C00G-H00G	119.9	C005-C00G-H00G	119.9
C00D-C00H-C00L	119.88(18)	C00D-C00H-H00H	120.1
C00L-C00H-H00H	120.1	N003-C00I-H00F	109.5
N003-C00I-H00I	109.5	H00F-C00I-H00I	109.5
N003-C00I-H00J	109.5	H00F-C00I-H00J	109.5
H00I-C00I-H00J	109.5	N003-C00J-H00K	109.5
N003-C00J-H00L	109.5	H00K-C00J-H00L	109.5

N003-C00J-H00M	109.5	H00K-C00J-H00M	109.5
H00L-C00J-H00M	109.5	C00L-C00K-C00B	120.26(18)
C00L-C00K-H00N	119.9	C00B-C00K-H00N	119.9
C00K-C00L-C00H	119.98(17)	C00K-C00L-H00O	120.0
C00H-C00L-H00O	120.0	C005-C004-C009	118.80(15)
C005-C004-C007	120.36(14)	C009-C004-C007	120.84(14)
C00G-C005-C004	120.36(15)	C00G-C005-H005	119.8
C004-C005-H005	119.8	C00D-C006-C00B	118.41(15)
C00D-C006-C008	120.97(14)	C00B-C006-C008	120.62(15)
C008-C007-C004	130.22(14)	C008-C007-C00A	110.14(14)
C004-C007-C00A	119.58(13)	C007-C008-C006	130.01(15)
C007-C008-C00F	107.67(14)	C006-C008-C00F	122.32(14)
C00C-C009-C004	120.52(16)	C00C-C009-H009	119.7
C004-C009-H009	119.7		

Table 6. Anisotropic atomic displacement parameters (Å^2) for **3aa**.

The anisotropic atomic displacement factor exponent takes the form: $-2\pi^2[h^2 a^{*2} U_{11} + ... + 2 h k a^* b^* U_{12}]$

	U_{11}	U_{22}	U_{33}	U_{23}	U_{13}	U_{12}
O001	0.0489(7)	0.0330(6)	0.0372(6)	0.0045(5)	0.0109(6)	-0.0041(6)
O002	0.0593(9)	0.0270(6)	0.0600(8)	0.0016(6)	0.0113(7)	-0.0086(6)
N003	0.0377(8)	0.0426(8)	0.0262(7)	0.0009(6)	0.0025(6)	0.0039(7)
C00A	0.0345(9)	0.0282(8)	0.0318(8)	0.0007(7)	0.0058(7)	0.0014(7)
C00B	0.0317(8)	0.0320(9)	0.0486(10)	-0.0072(8)	0.0018(8)	-0.0006(7)

C00C	0.0487(10)	0.0245(8)	0.0396(9)	-0.0047(7)	-0.0135(8)	0.0065(8)
C00D	0.0330(8)	0.0315(9)	0.0365(8)	-0.0048(7)	-0.0023(7)	-0.0042(7)
C00E	0.0505(11)	0.0259(8)	0.0355(8)	0.0051(7)	-0.0121(8)	-0.0061(8)
C00F	0.0364(9)	0.0299(9)	0.0417(9)	0.0024(7)	0.0071(8)	-0.0011(7)
C00G	0.0383(9)	0.0343(8)	0.0280(8)	0.0028(7)	-0.0037(7)	-0.0064(8)
C00H	0.0372(10)	0.0487(10)	0.0354(9)	-0.0042(8)	-0.0021(8)	-0.0081(9)
C00I	0.0558(12)	0.0472(10)	0.0274(8)	0.0011(8)	0.0050(8)	0.0057(9)
C00J	0.0396(10)	0.0720(14)	0.0334(9)	0.0039(9)	-0.0017(8)	0.0120(10)
C00K	0.0334(9)	0.0450(10)	0.0565(12)	-0.0209(9)	0.0052(9)	0.0001(8)
C00L	0.0341(9)	0.0622(12)	0.0388(9)	-0.0177(9)	0.0053(8)	-0.0100(9)
C004	0.0294(8)	0.0243(7)	0.0252(7)	-0.0023(6)	-0.0044(6)	0.0013(6)
C005	0.0329(9)	0.0273(8)	0.0266(7)	-0.0004(6)	-0.0014(7)	0.0002(7)
C006	0.0251(7)	0.0286(8)	0.0367(8)	-0.0058(7)	0.0015(7)	-0.0039(7)
C007	0.0252(8)	0.0284(8)	0.0293(8)	-0.0010(6)	0.0009(7)	0.0026(6)
C008	0.0263(8)	0.0255(8)	0.0370(8)	0.0011(7)	0.0020(7)	-0.0003(6)
C009	0.0353(9)	0.0301(8)	0.0318(8)	-0.0059(7)	-0.0050(7)	0.0036(7)

Table 7. Hydrogen atomic coordinates and isotropic atomic displacement parameters (Å^2) for **3aa**.

	x/a	y/b	z/c	U(eq)
H00A	0.0685	0.3976	0.7412	0.038
H00B	0.3091	0.0315	0.5373	0.045
H00C	0.1847	0.8021	0.6222	0.045

H00D	0.1376	0.3853	0.4733	0.04
H00E	0.4014	0.8120	0.5396	0.045
H00G	0.5372	0.6218	0.5087	0.04
H00H	0.1873	0.3381	0.3397	0.049
H00F	0.1343	0.4094	0.8759	0.065
H00I	0.3118	0.4062	0.9022	0.065
H00J	0.2244	0.2729	0.8805	0.065
H00K	0.4589	0.3370	0.7128	0.073
H00L	0.4250	0.2278	0.7790	0.073
H00M	0.5074	0.3619	0.8037	0.073
H00N	0.3532	-0.0170	0.4027	0.054
H00O	0.2932	0.1359	0.3042	0.054
H005	0.4474	0.4189	0.5525	0.035
H009	0.0995	0.6007	0.6708	0.039

2. ^{1}H and ^{13}C NMR spectra data

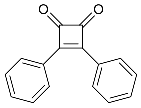

^{1}H NMR, $CDCl_3$, 500 MHz, **1a**

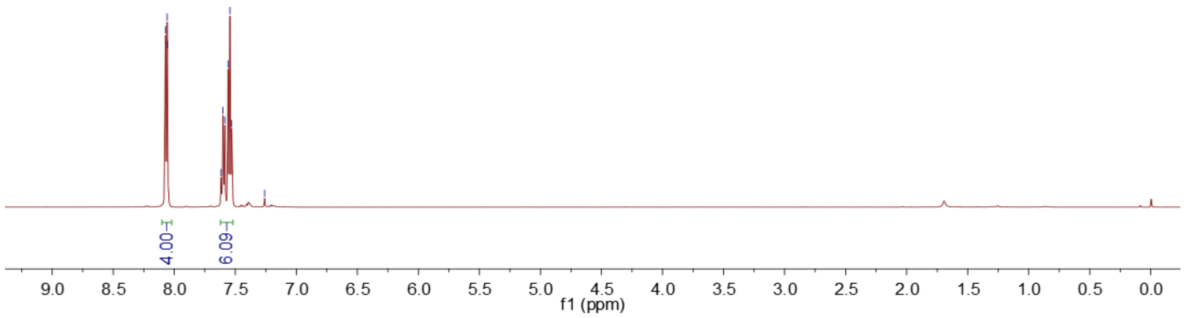

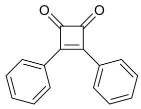

^{13}C NMR, $CDCl_3$, 125 MHz, **1a**

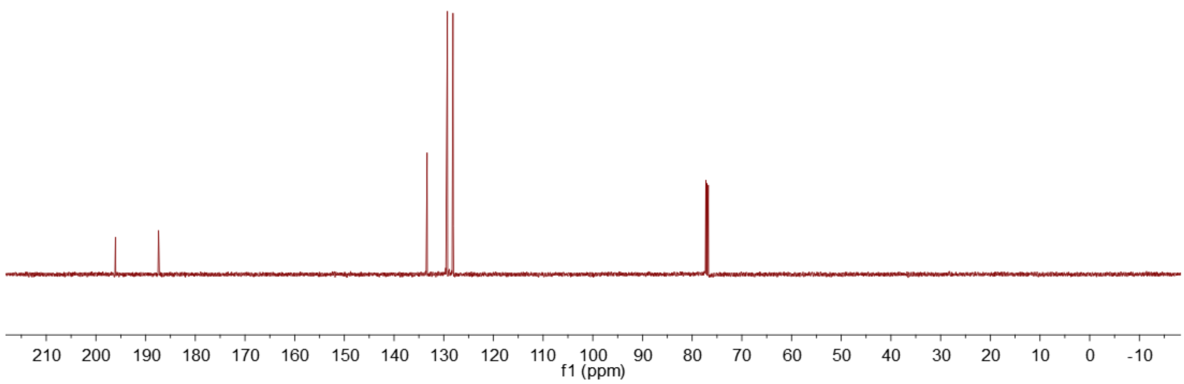

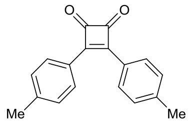

^{1}H NMR, $CDCl_3$, 500 MHz, **1b**

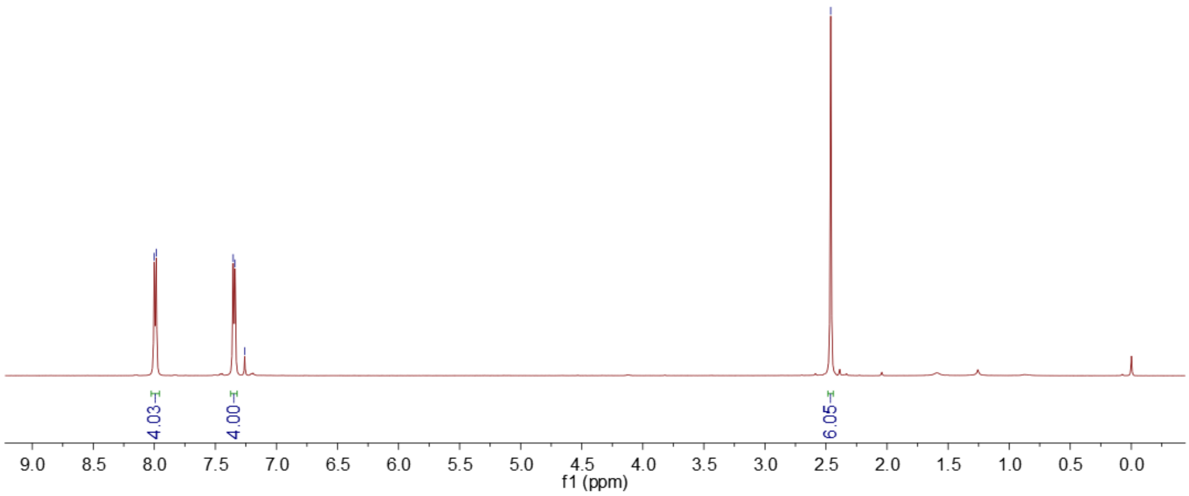

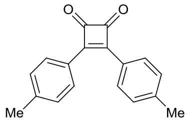

^{13}C NMR, $CDCl_3$, 125 MHz, **1b**

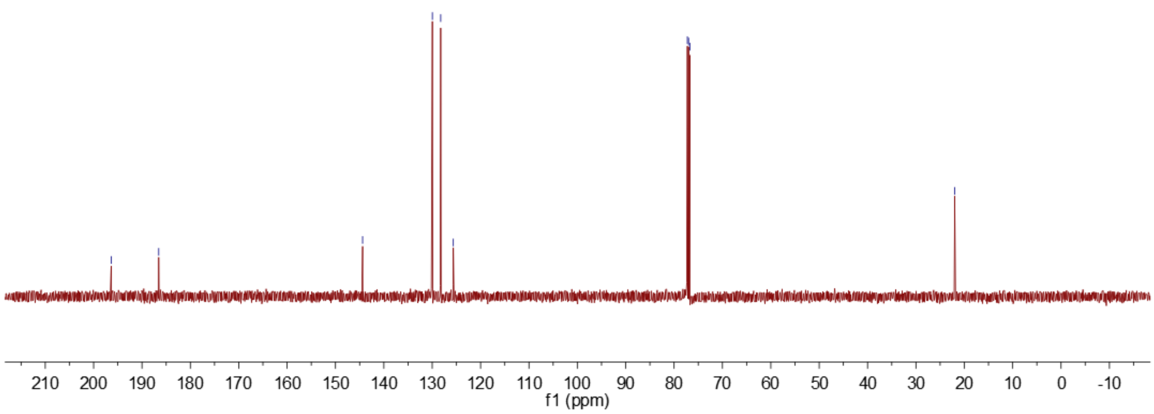

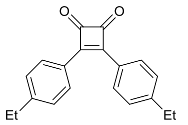

^{1}H NMR, $CDCl_3$, 500 MHz, **1c**

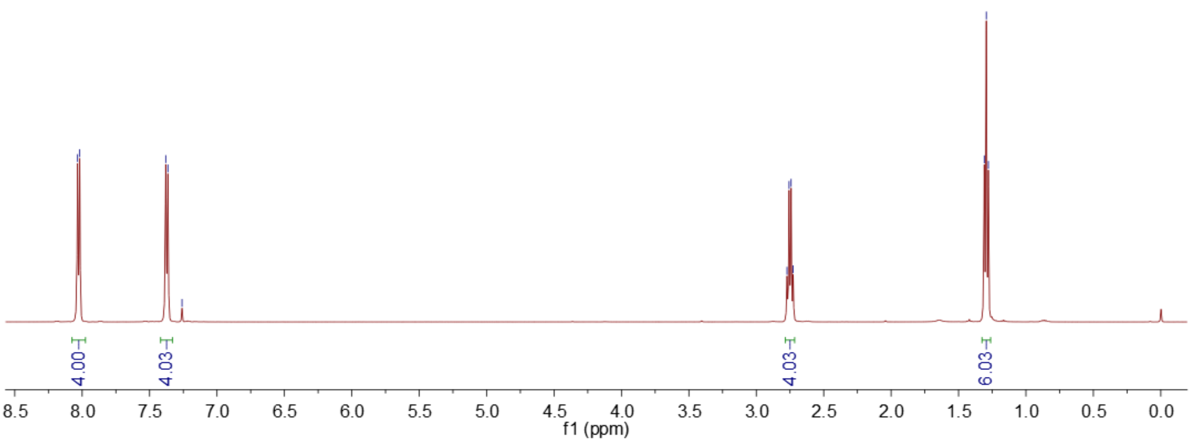

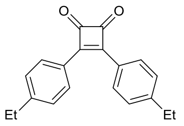

^{13}C NMR, $CDCl_3$, 125 MHz, **1c**

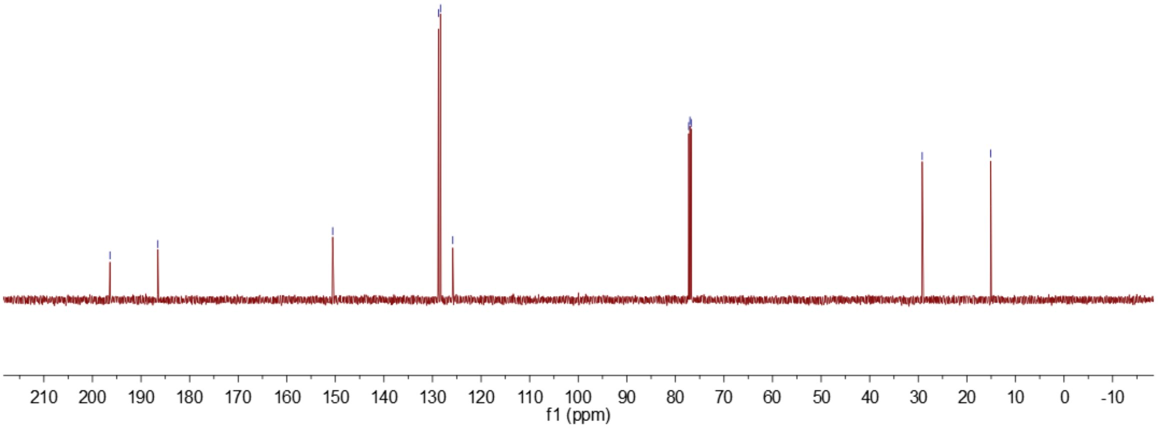

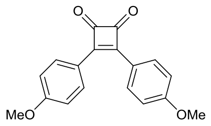

^{1}H NMR, $CDCl_3$, 500 MHz, **1d**

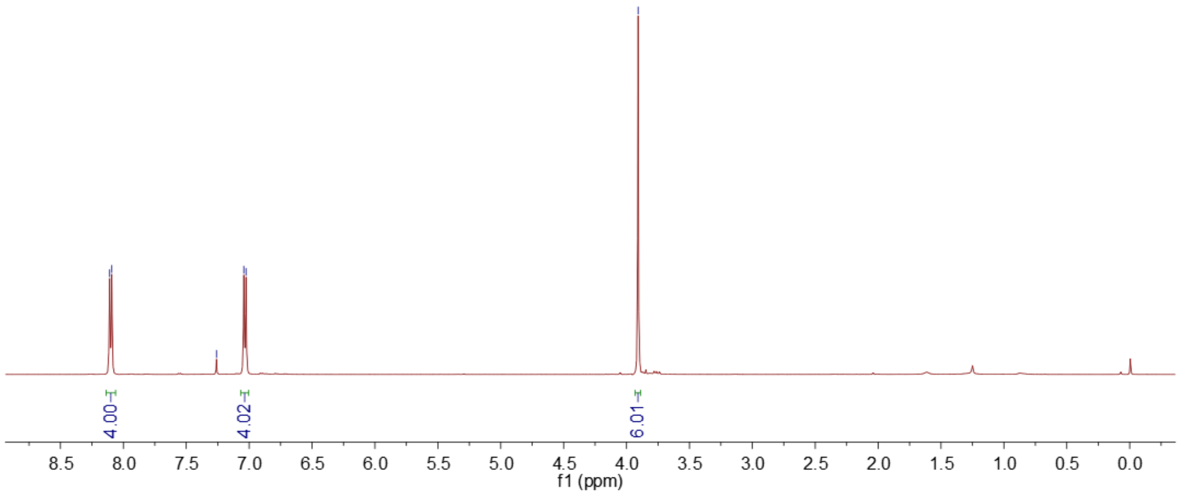

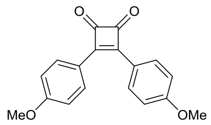

^{13}C NMR, $CDCl_3$, 125 MHz, **1d**

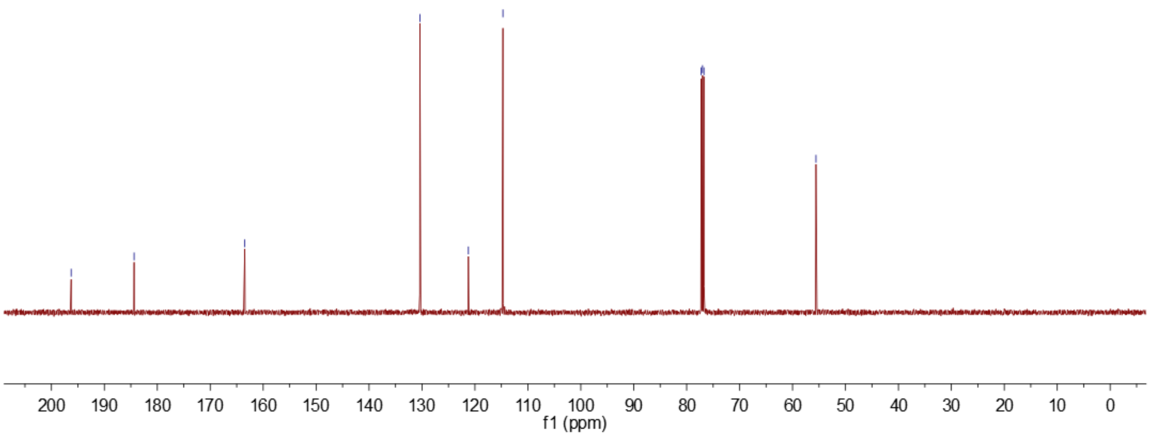

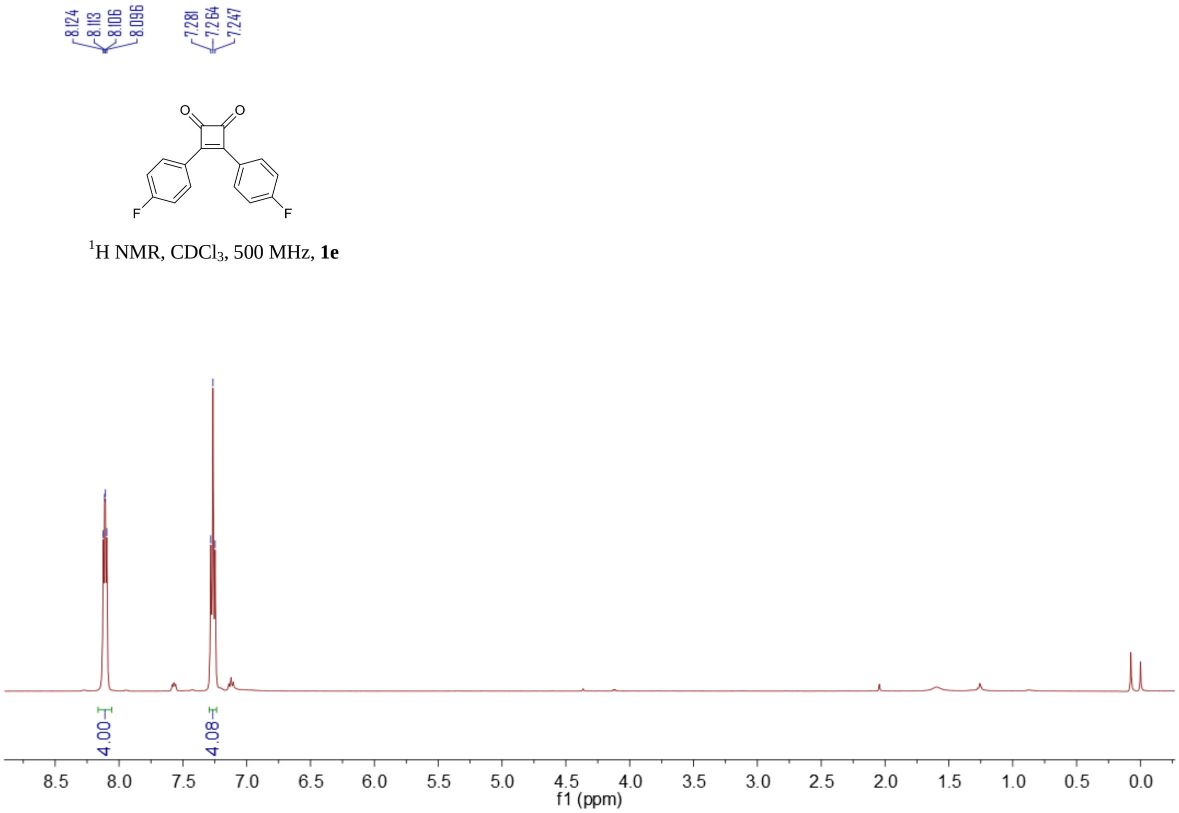

^{1}H NMR, $CDCl_3$, 500 MHz, **1e**

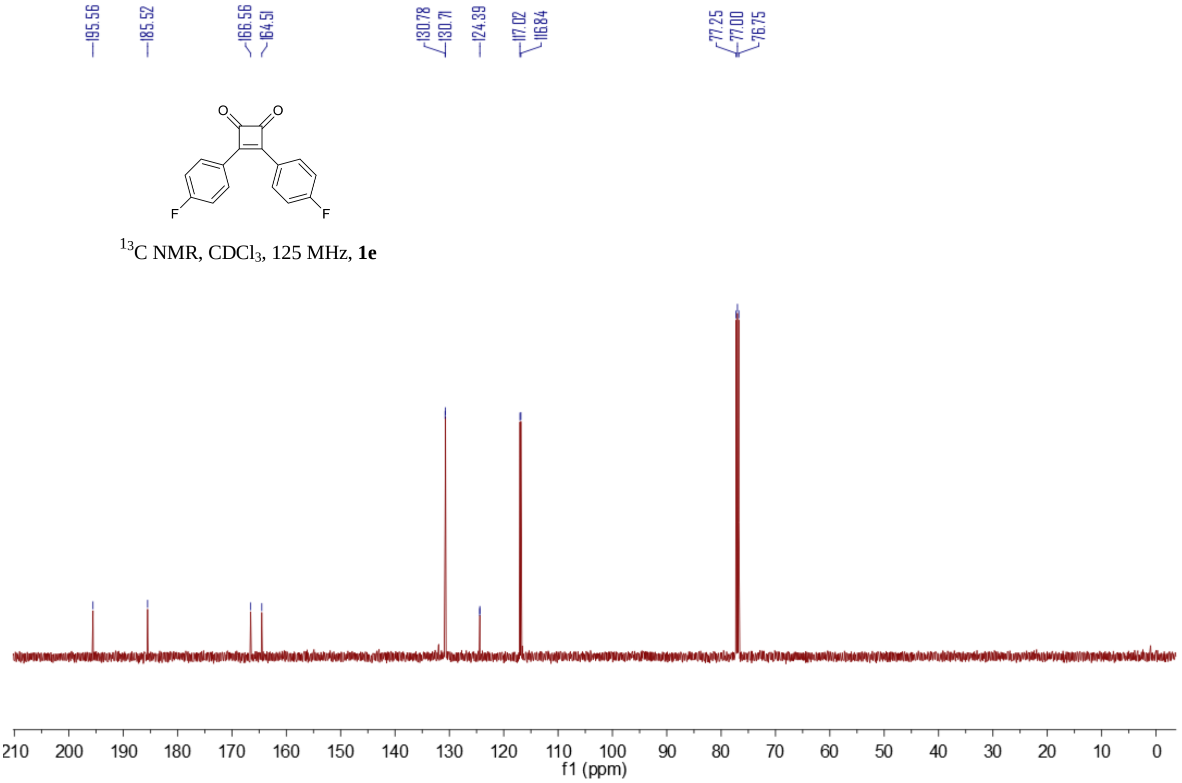

^{13}C NMR, $CDCl_3$, 125 MHz, **1e**

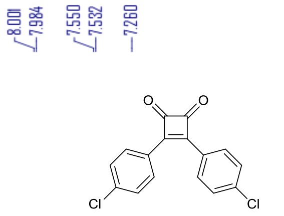

^{1}H NMR, $CDCl_3$, 500 MHz, **1f**

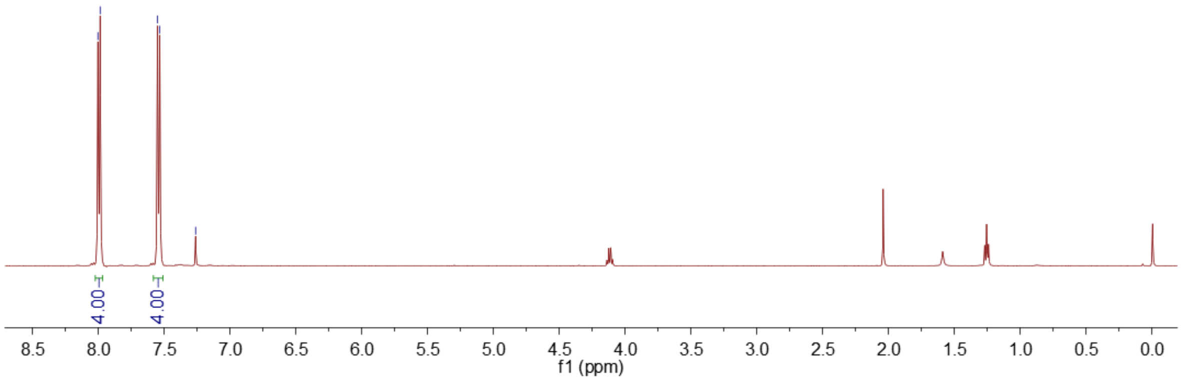

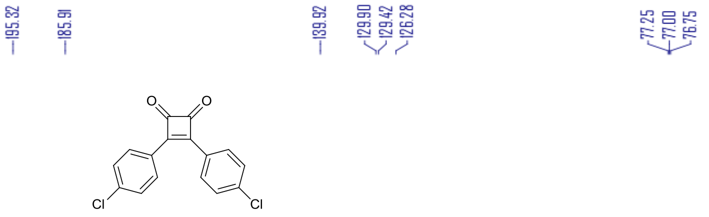

^{13}C NMR, $CDCl_3$, 125 MHz, **1f**

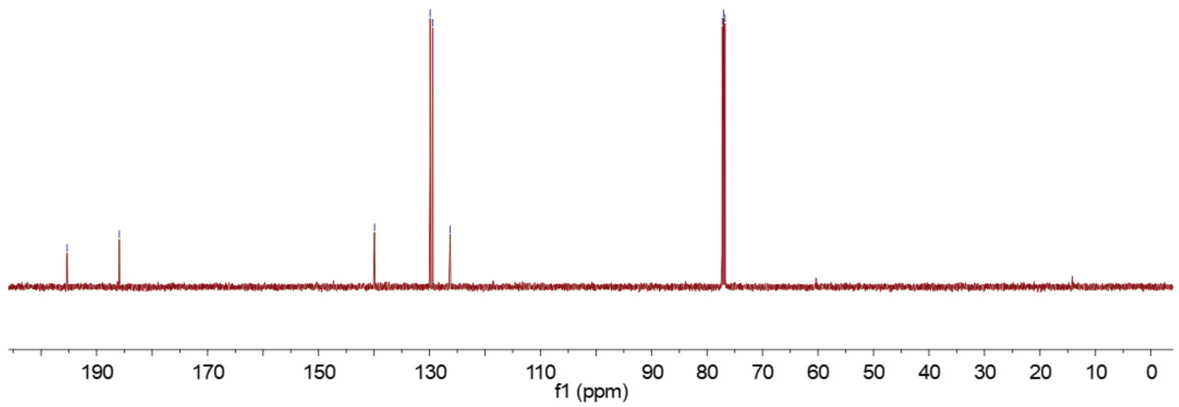

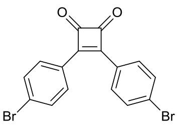

^{1}H NMR, $CDCl_3$, 500 MHz, **1g**

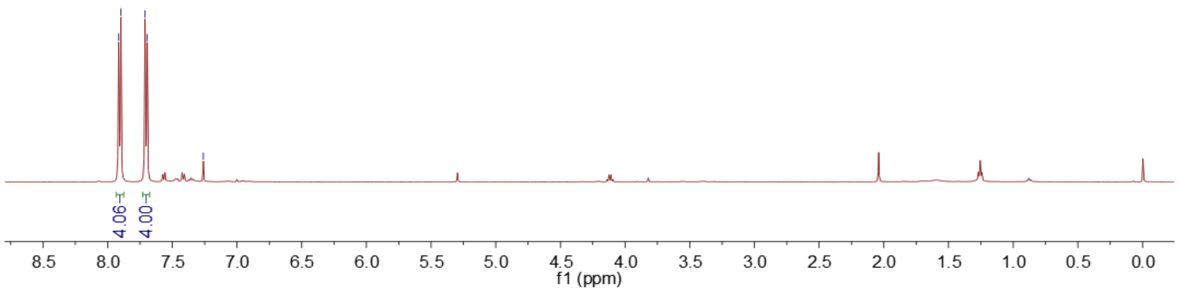

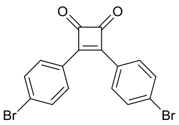

^{13}C NMR, $CDCl_3$, 125 MHz, **1g**

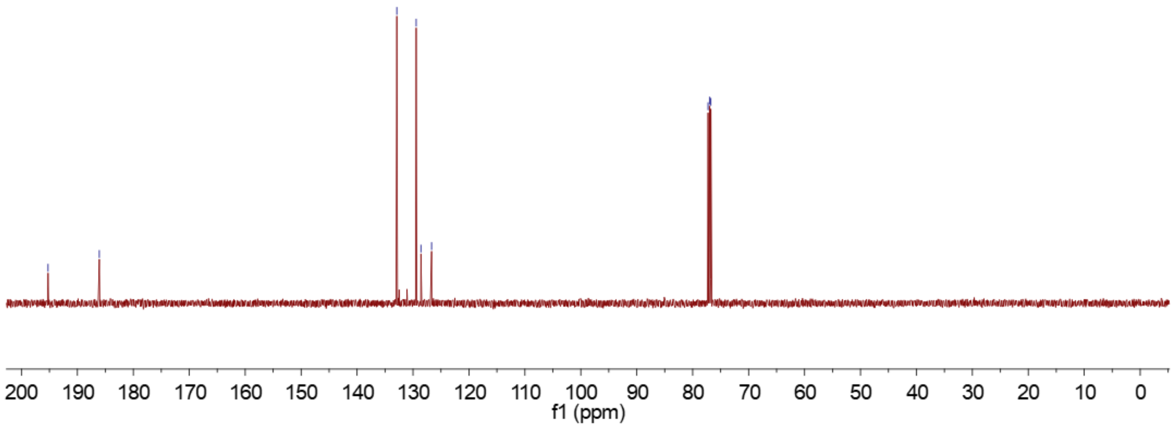

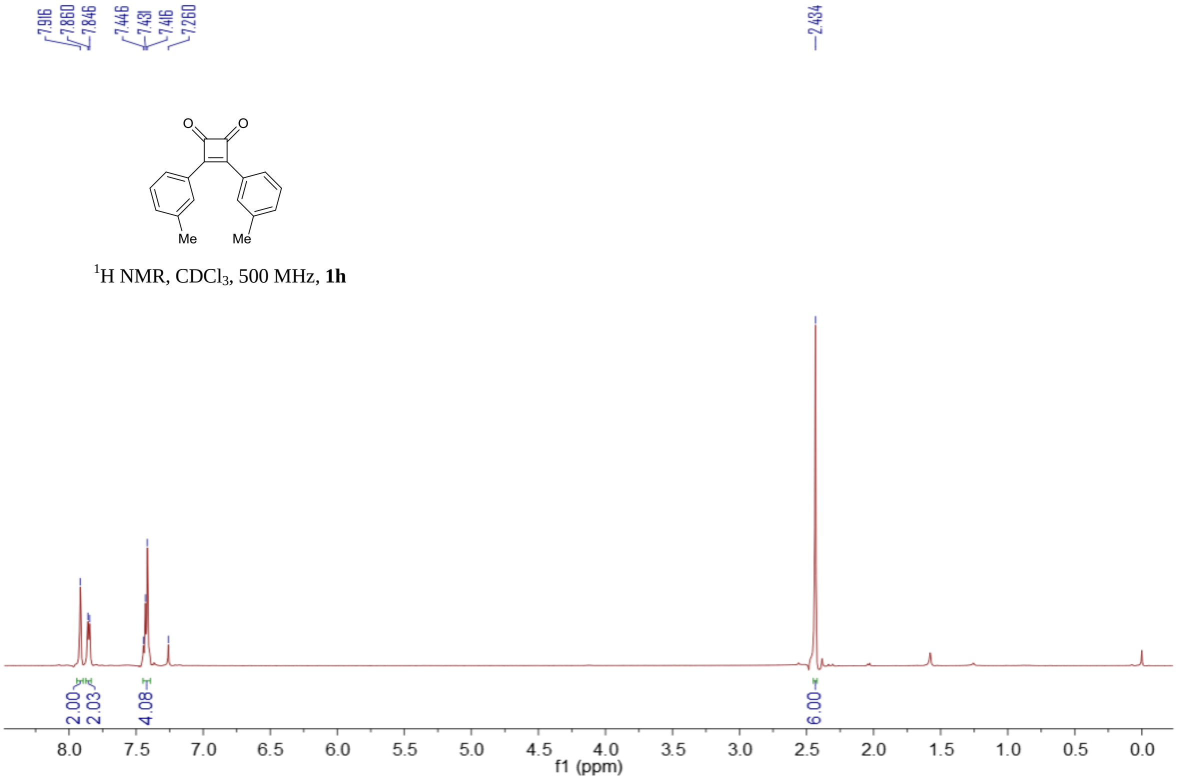

^{1}H NMR, $CDCl_3$, 500 MHz, **1h**

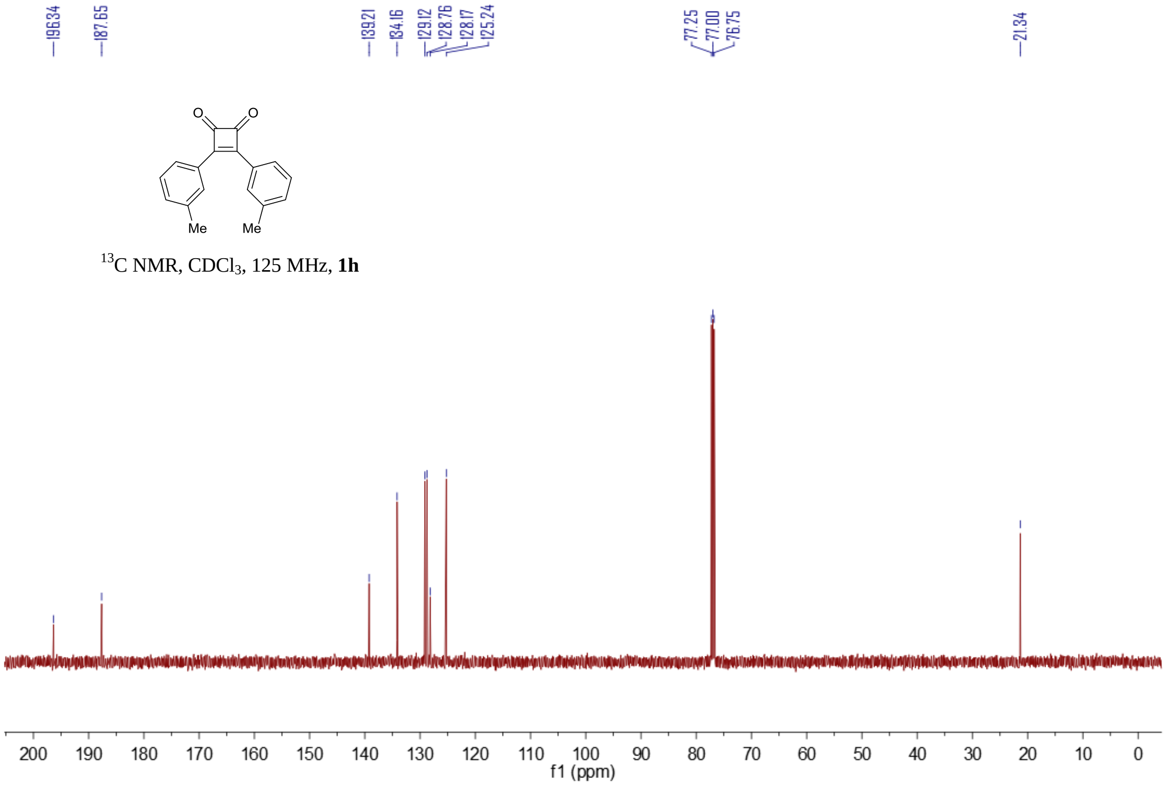

^{13}C NMR, $CDCl_3$, 125 MHz, **1h**

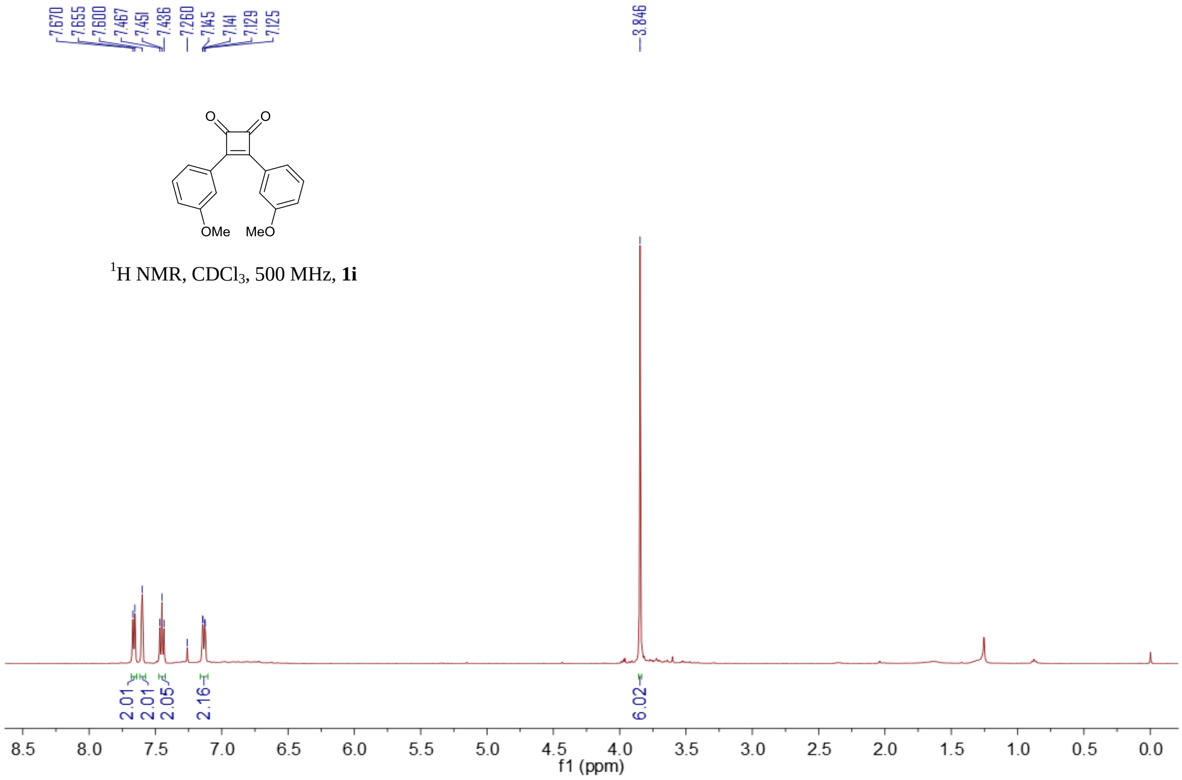

^{1}H NMR, $CDCl_3$, 500 MHz, **1i**

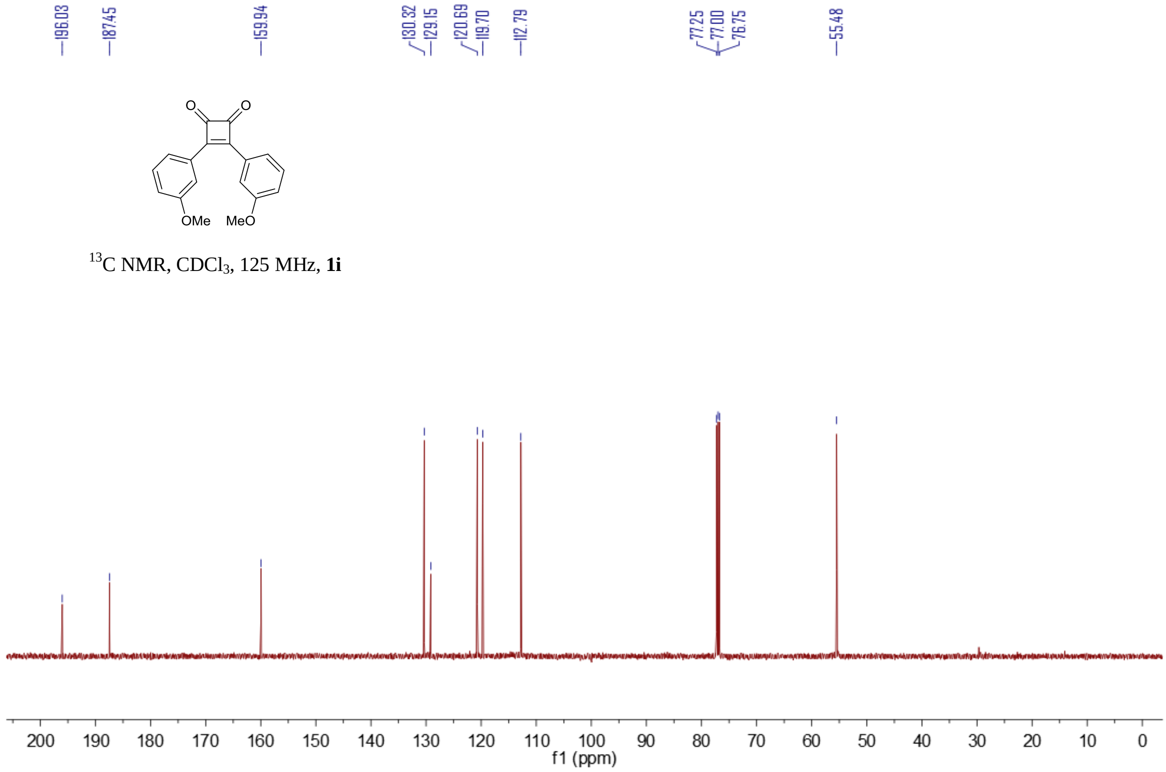

^{13}C NMR, $CDCl_3$, 125 MHz, **1i**

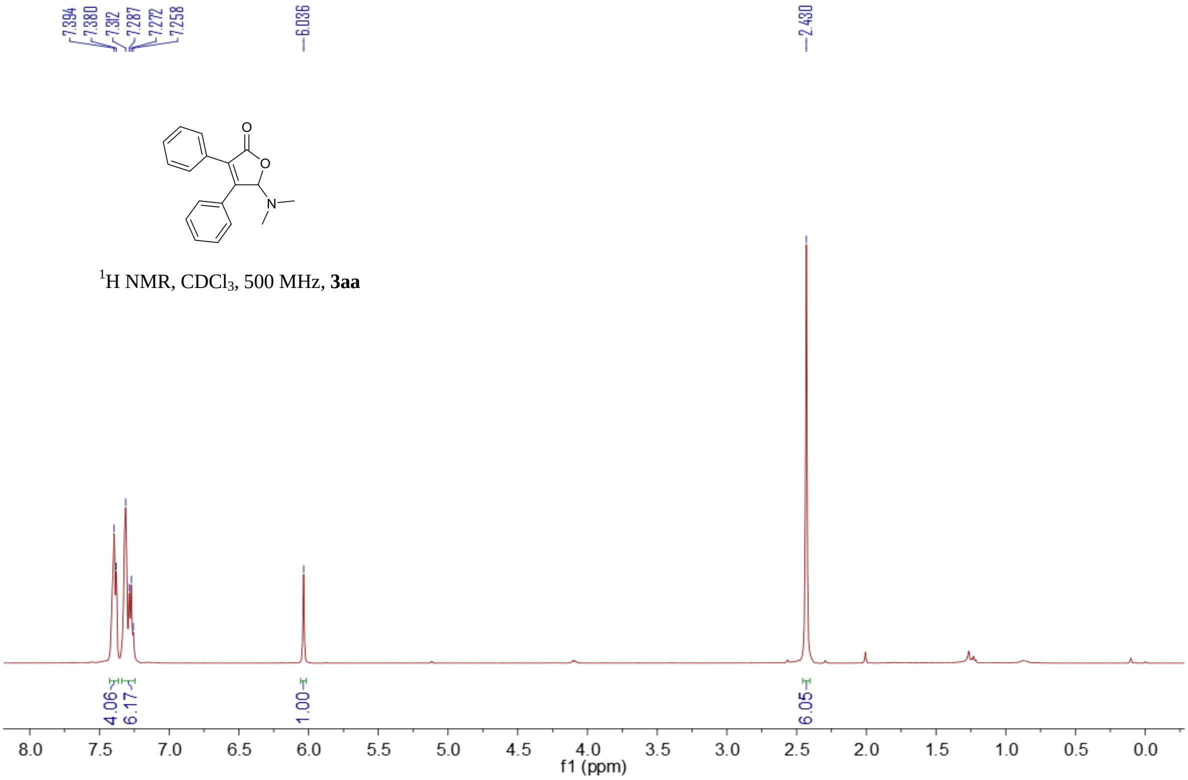

^{1}H NMR, $CDCl_3$, 500 MHz, **3aa**

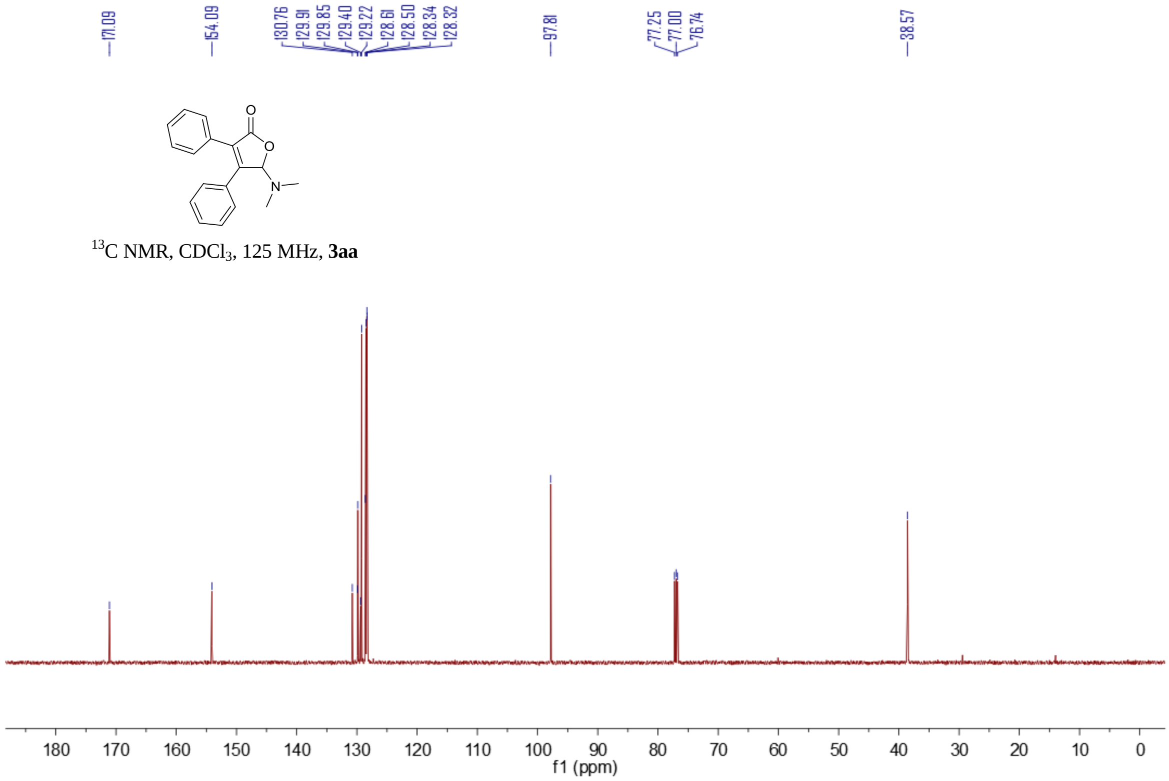

^{13}C NMR, $CDCl_3$, 125 MHz, **3aa**

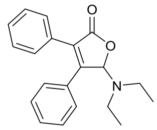

^{1}H NMR, $CDCl_3$, 500 MHz, **3ab**

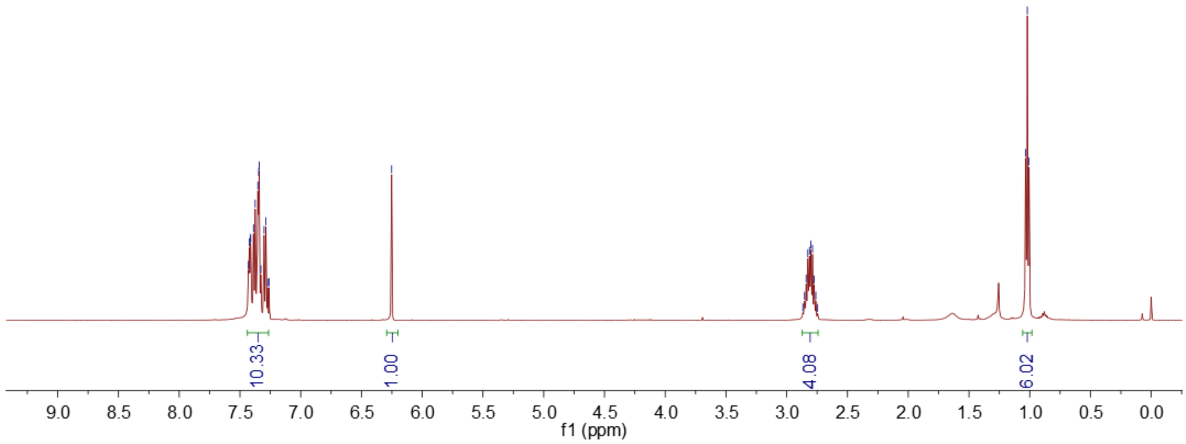

^{13}C NMR, $CDCl_3$, 125 MHz, **3ab**

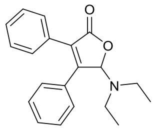

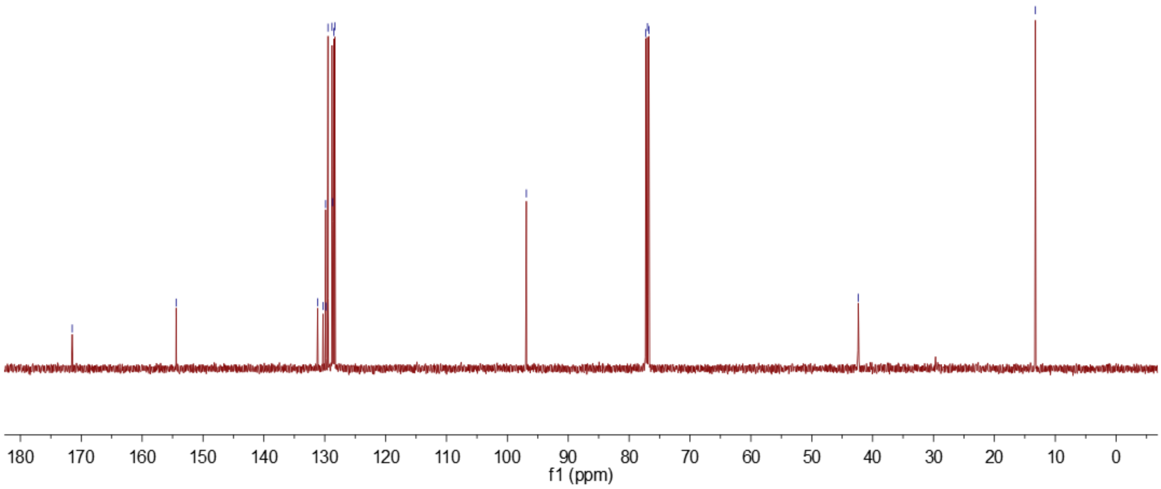

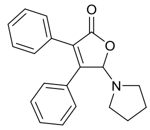

^{1}H NMR, $CDCl_3$, 500 MHz, **3ad**

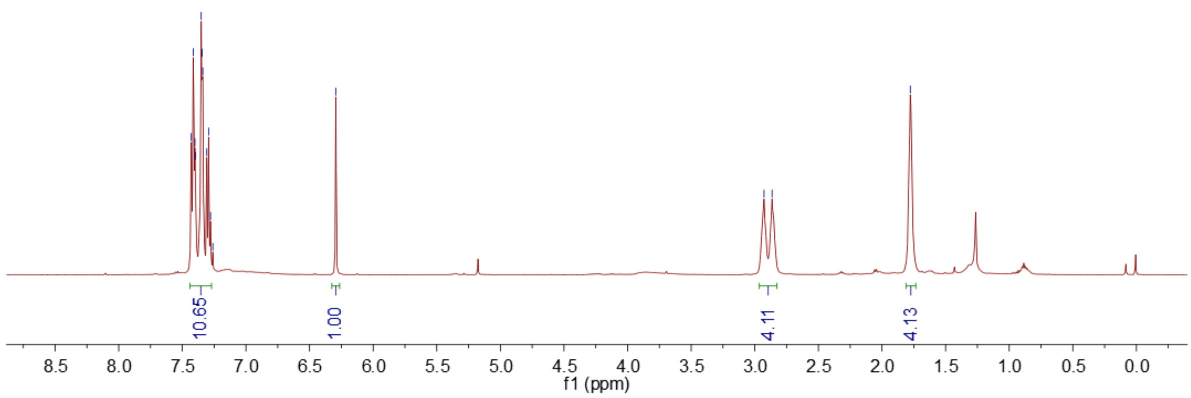

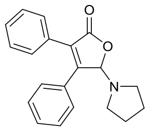

^{13}C NMR, $CDCl_3$, 125 MHz, **3ad**

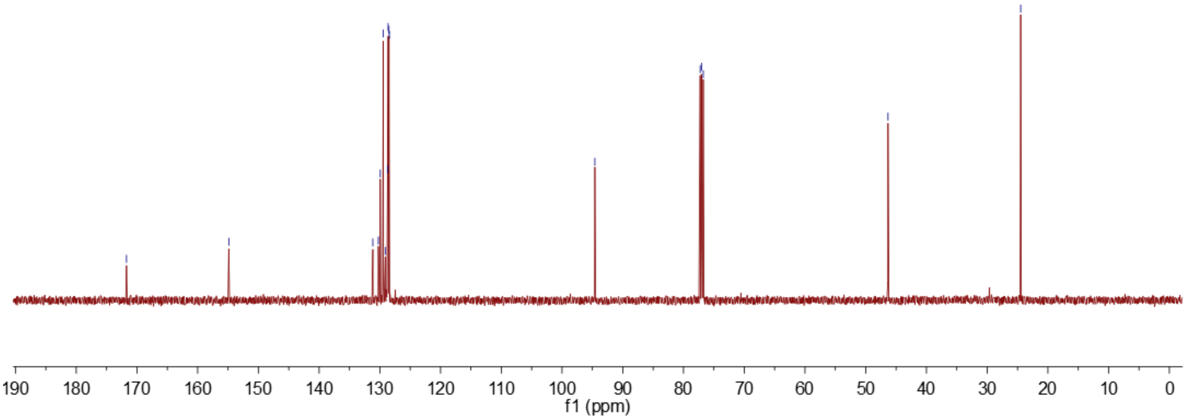

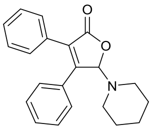

^{1}H NMR, $CDCl_3$, 500 MHz, **3ae**

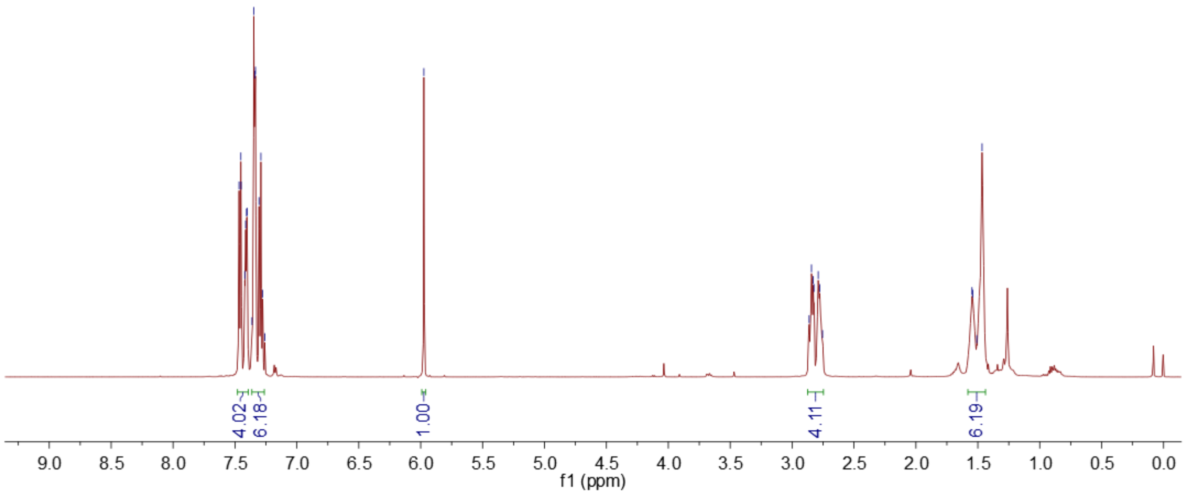

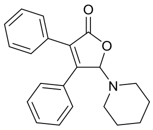

^{13}C NMR, $CDCl_3$, 125 MHz, **3ae**

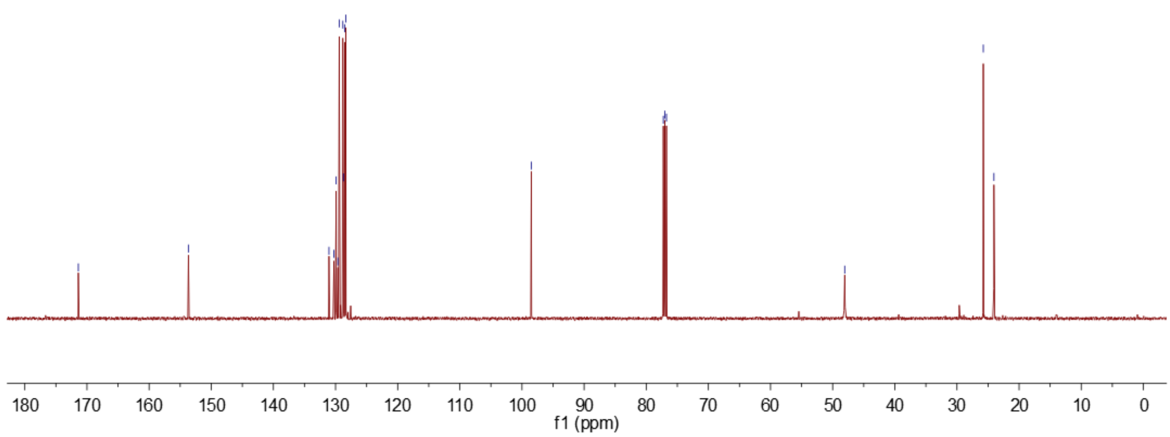

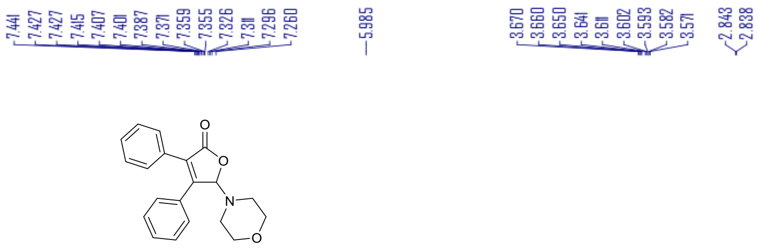

^{1}H NMR, $CDCl_3$, 500 MHz, **3af**

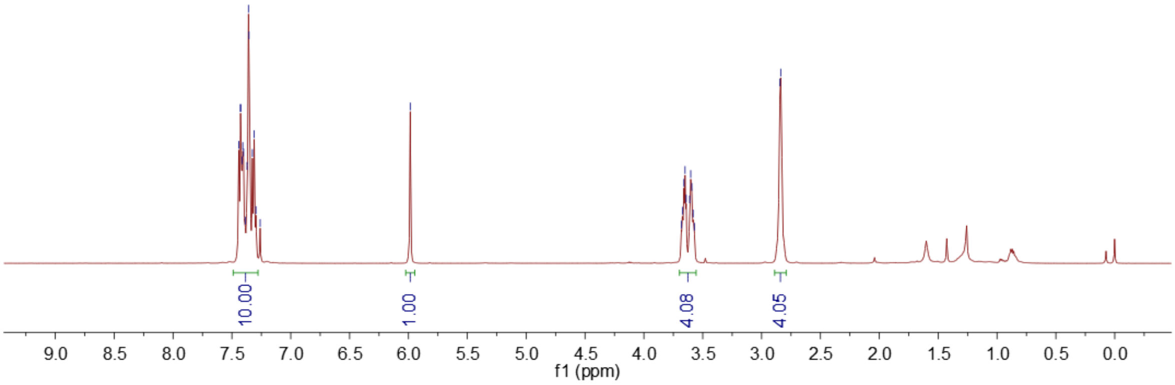

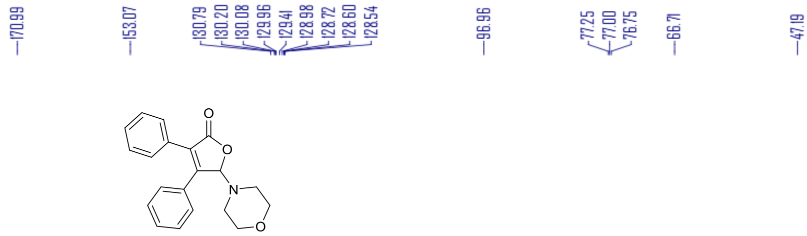

^{13}C NMR, $CDCl_3$, 125 MHz, **3af**

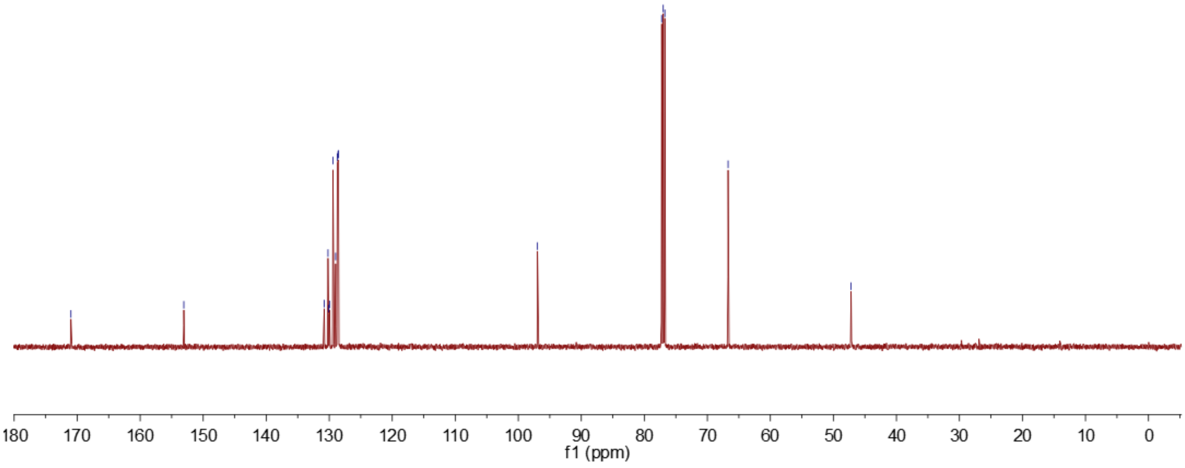

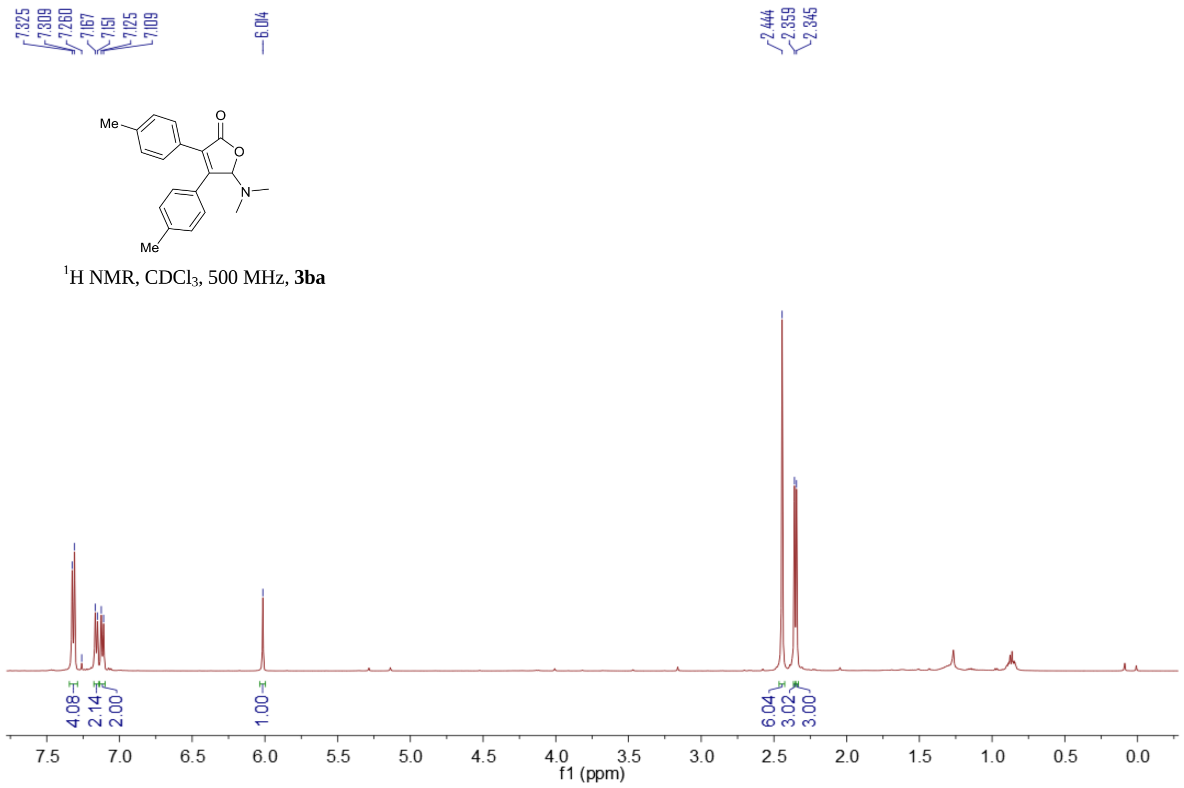

^{1}H NMR, $CDCl_3$, 500 MHz, **3ba**

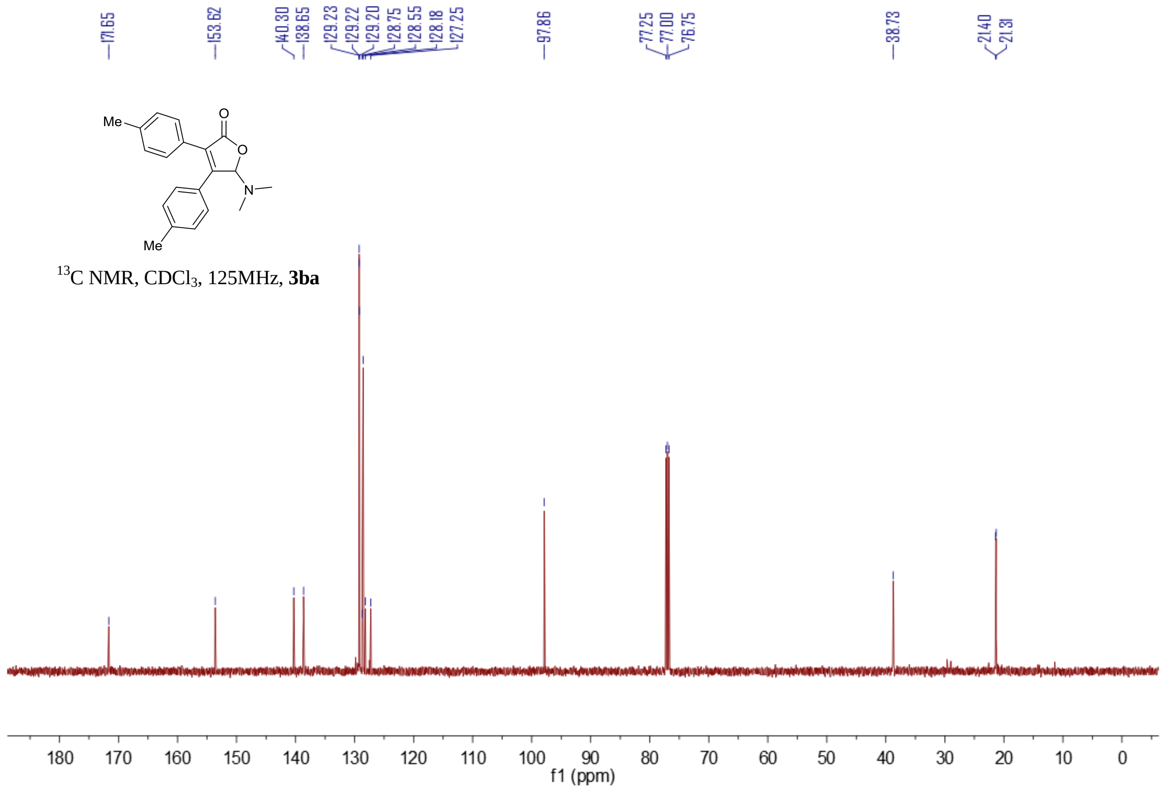

^{13}C NMR, $CDCl_3$, 125MHz, **3ba**

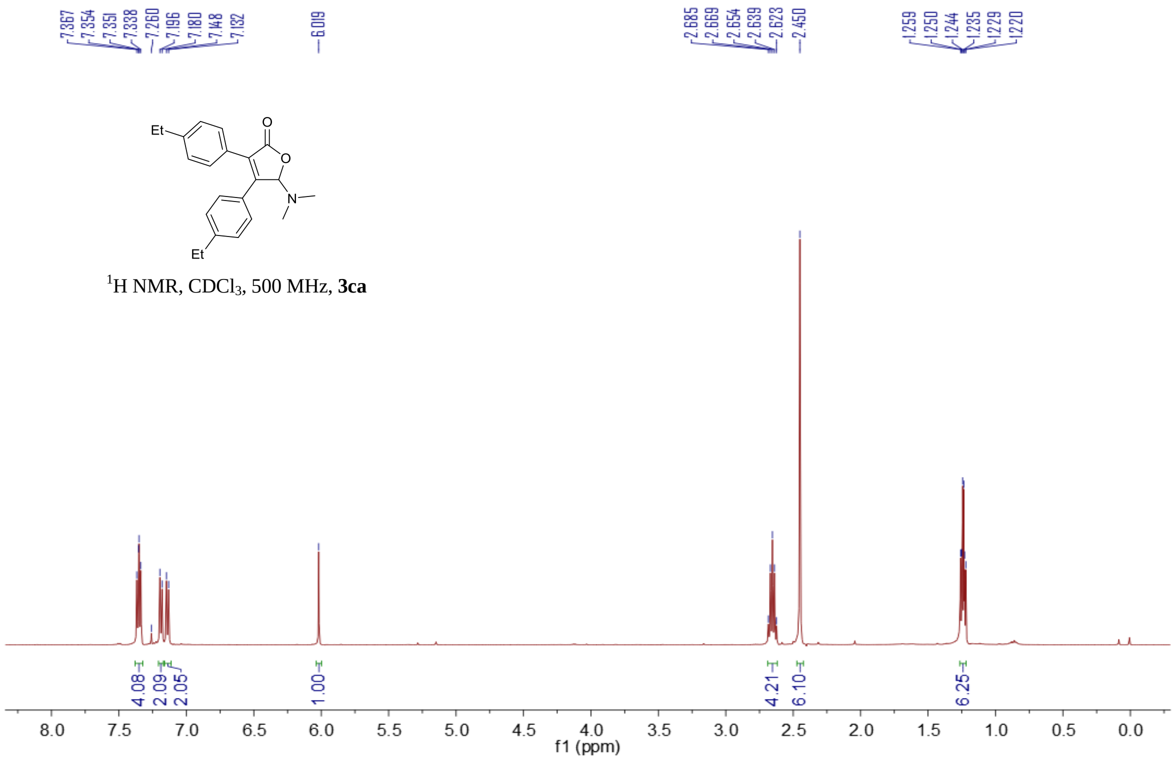

^{1}H NMR, $CDCl_3$, 500 MHz, **3ca**

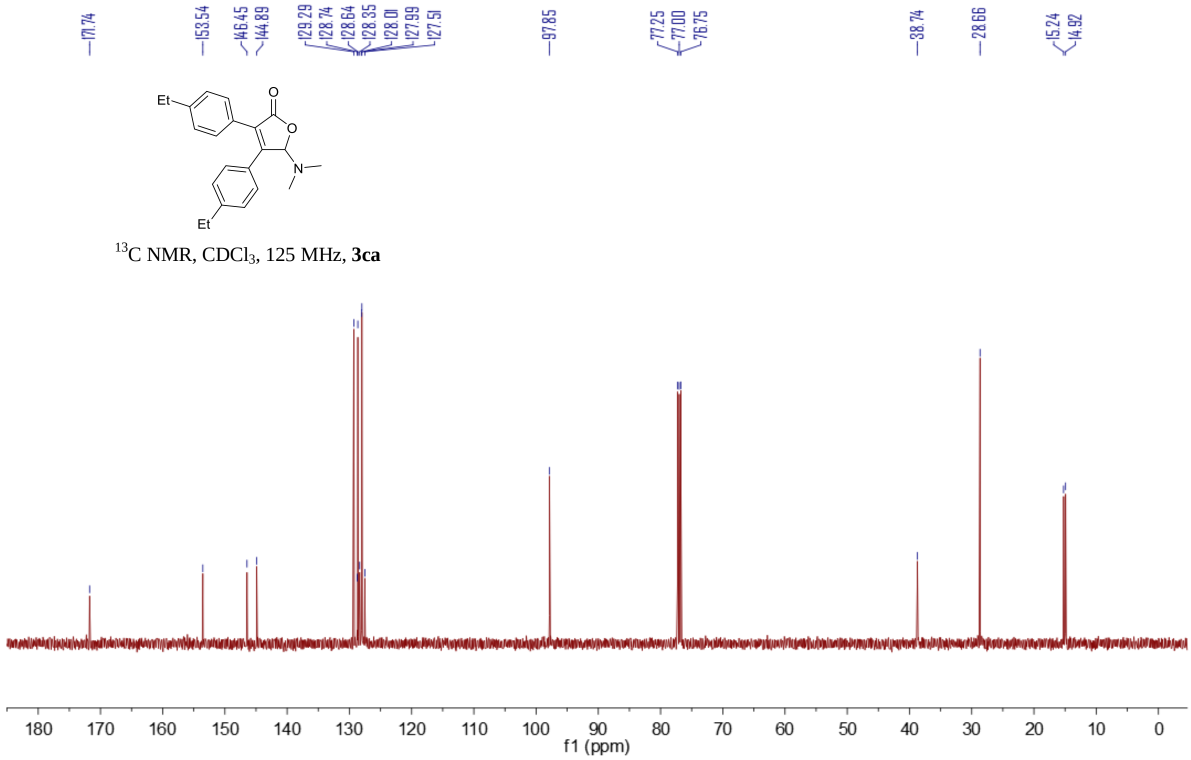

^{13}C NMR, $CDCl_3$, 125 MHz, **3ca**

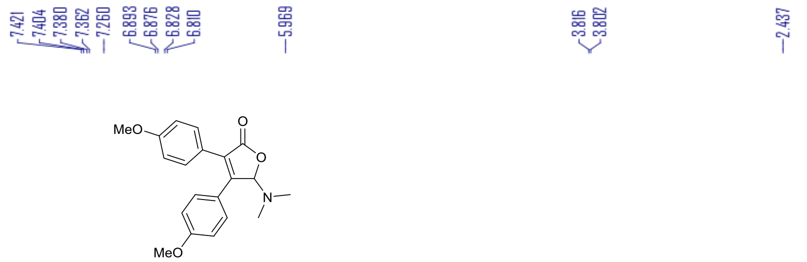

^{1}H NMR, $CDCl_3$, 500 MHz, **3da**

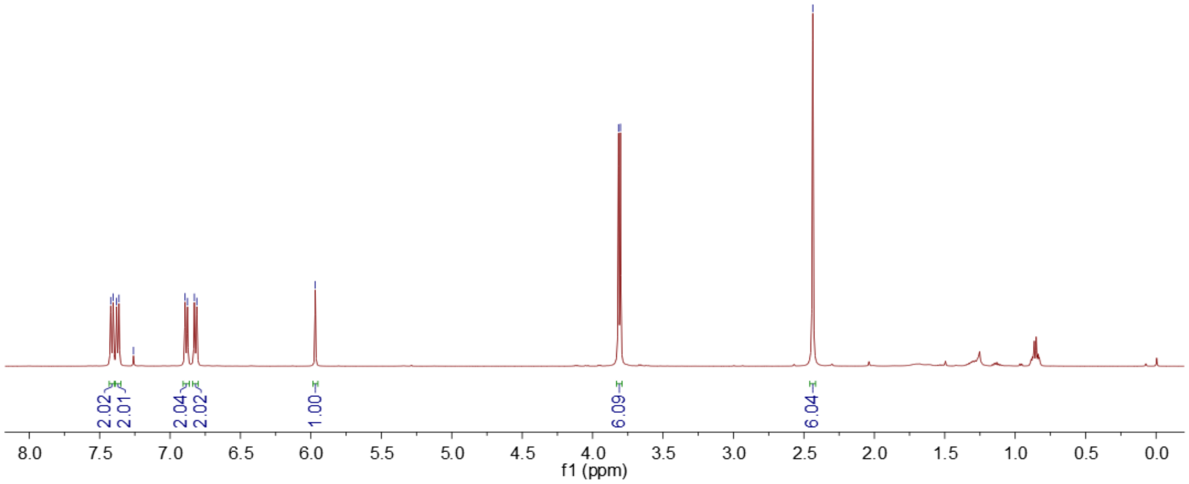

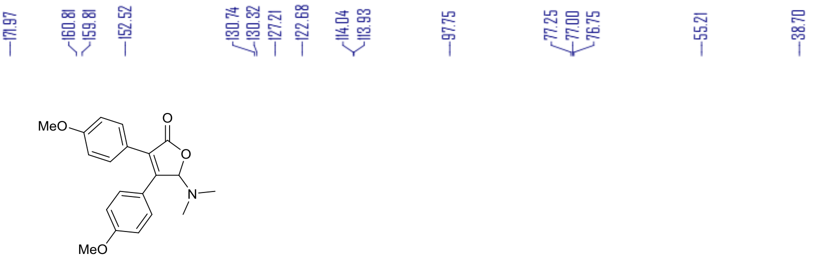

^{13}C NMR, $CDCl_3$, 125 MHz, **3da**

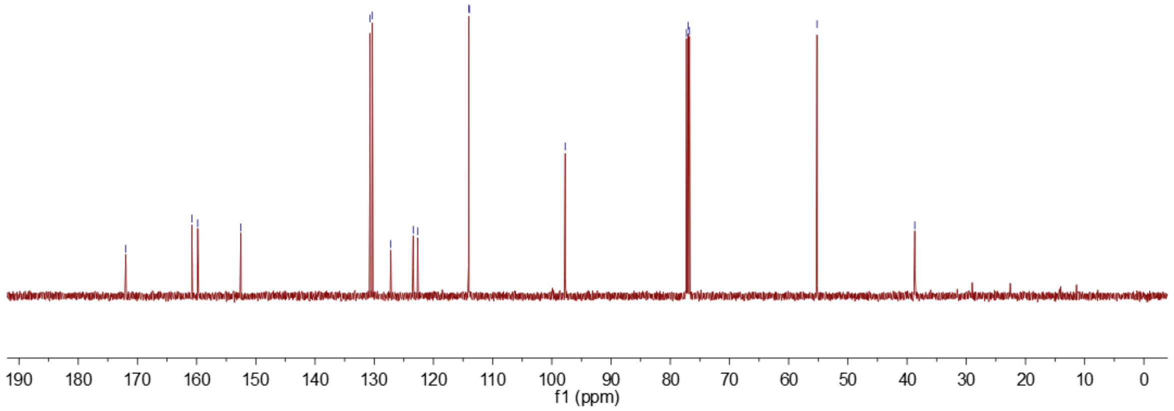

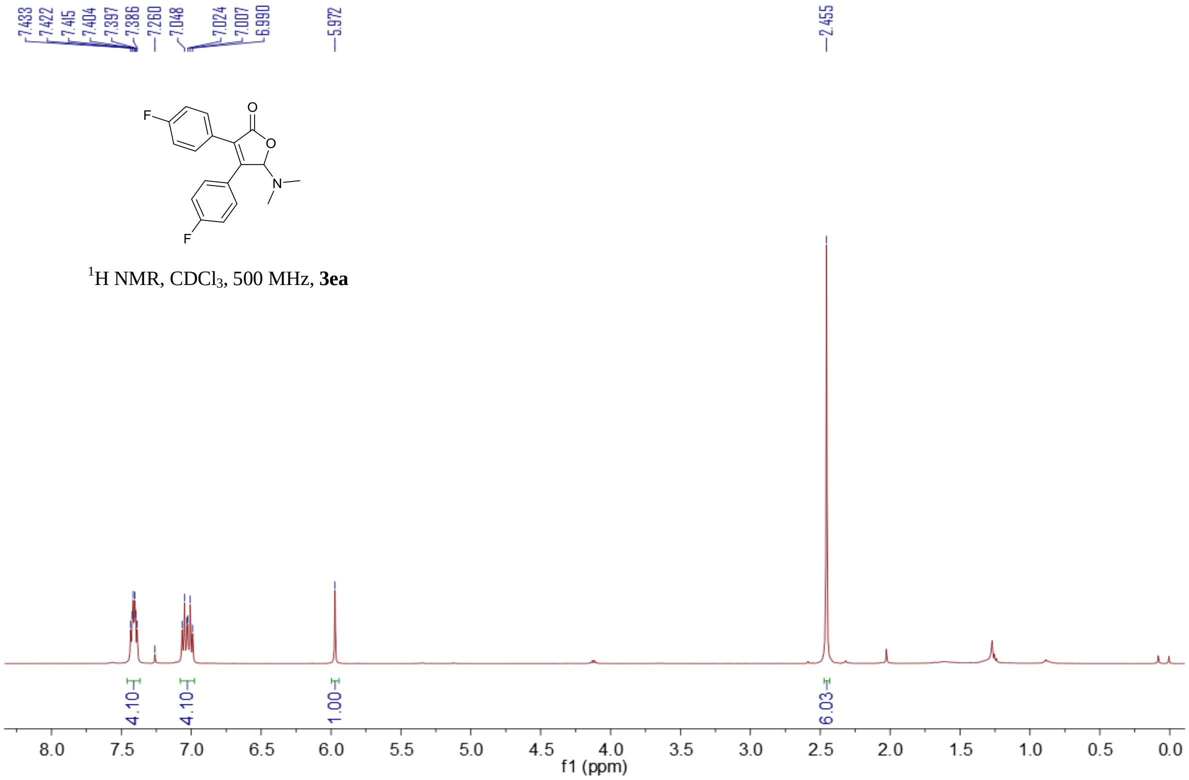

^{1}H NMR, $CDCl_3$, 500 MHz, **3ea**

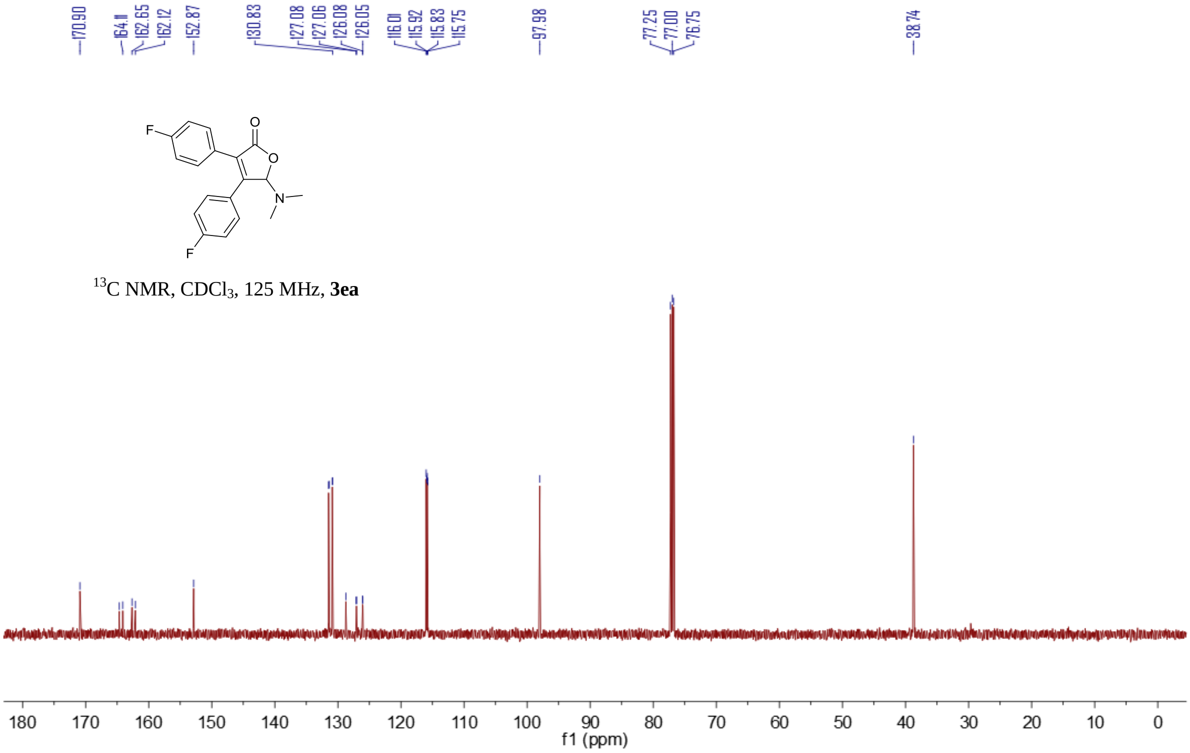

^{13}C NMR, $CDCl_3$, 125 MHz, **3ea**

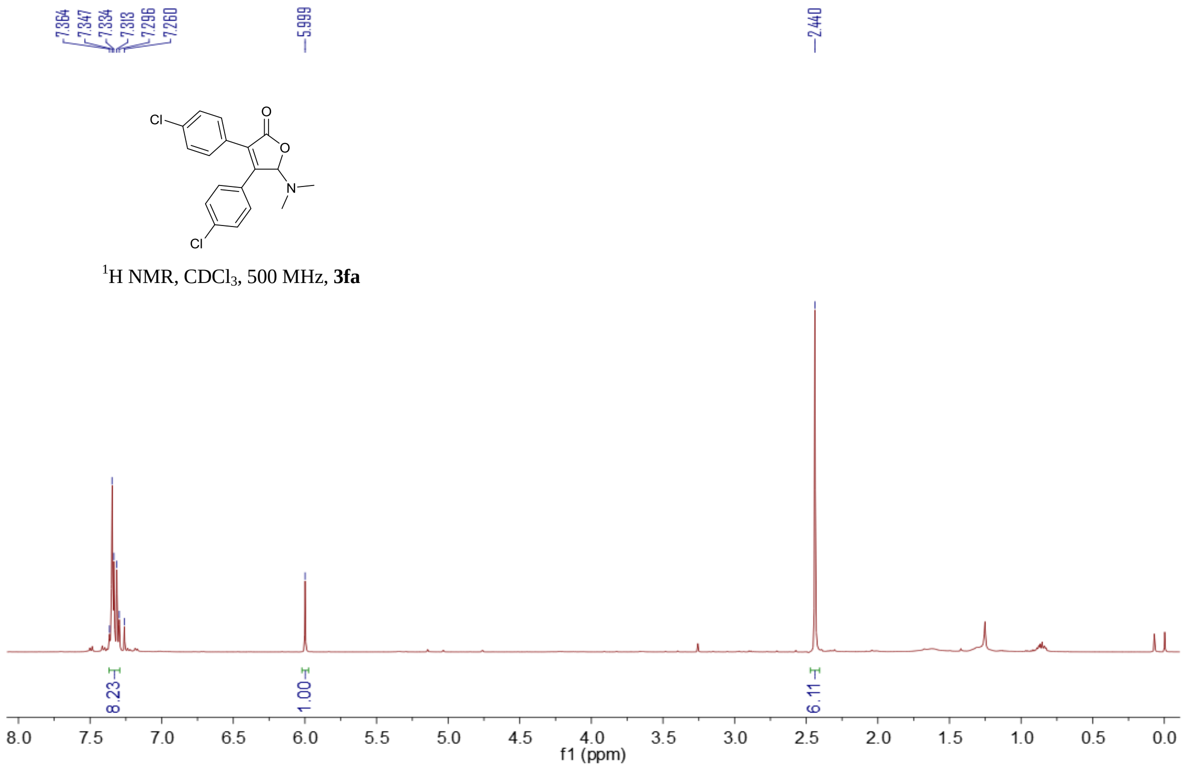

^{1}H NMR, $CDCl_3$, 500 MHz, **3fa**

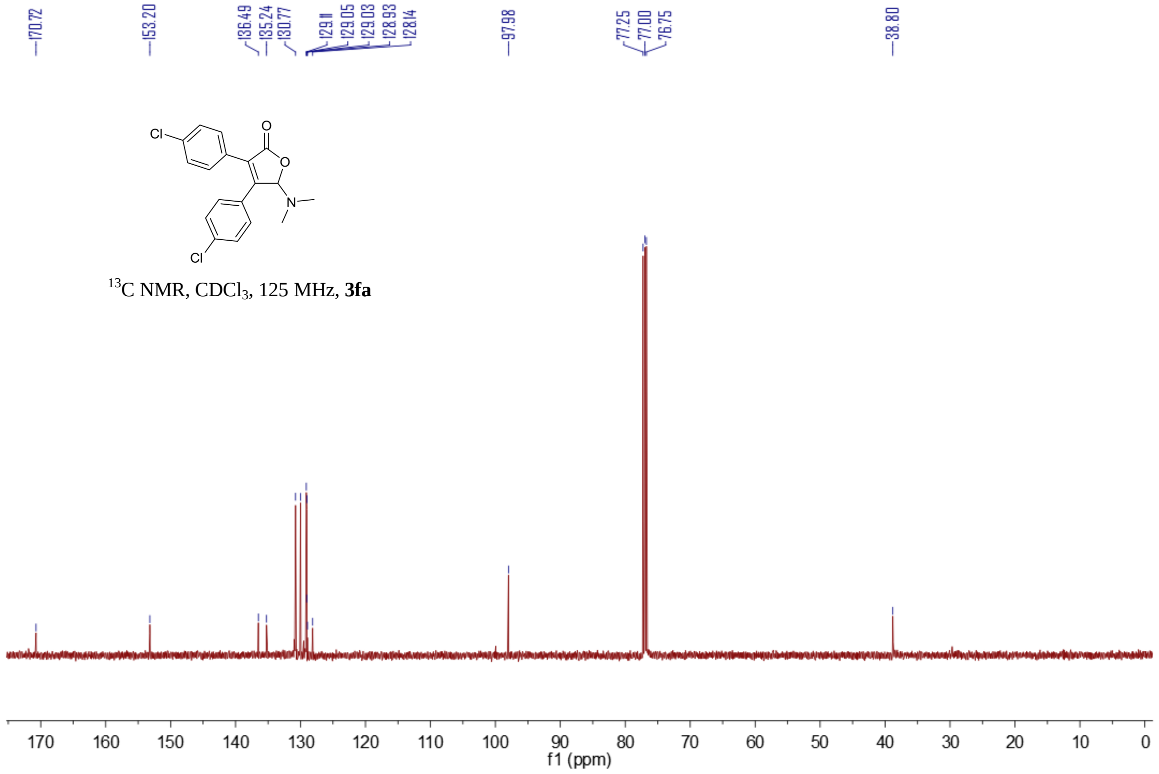

^{13}C NMR, $CDCl_3$, 125 MHz, **3fa**

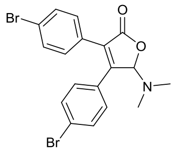

^{1}H NMR, $CDCl_3$, 500 MHz, **3ga**

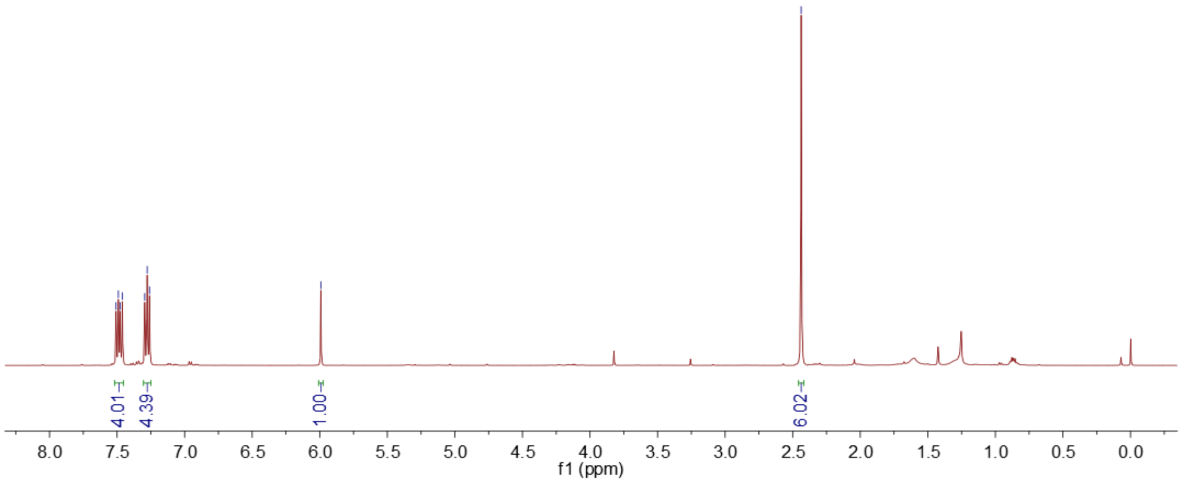

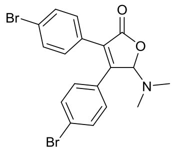

^{13}C NMR, $CDCl_3$, 125 MHz, **3ga**

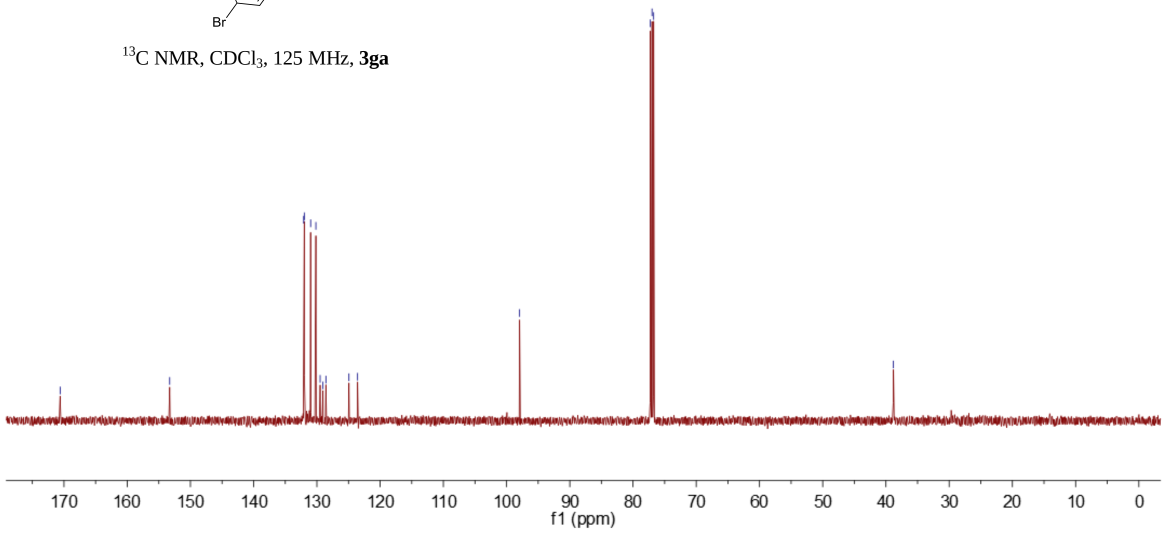

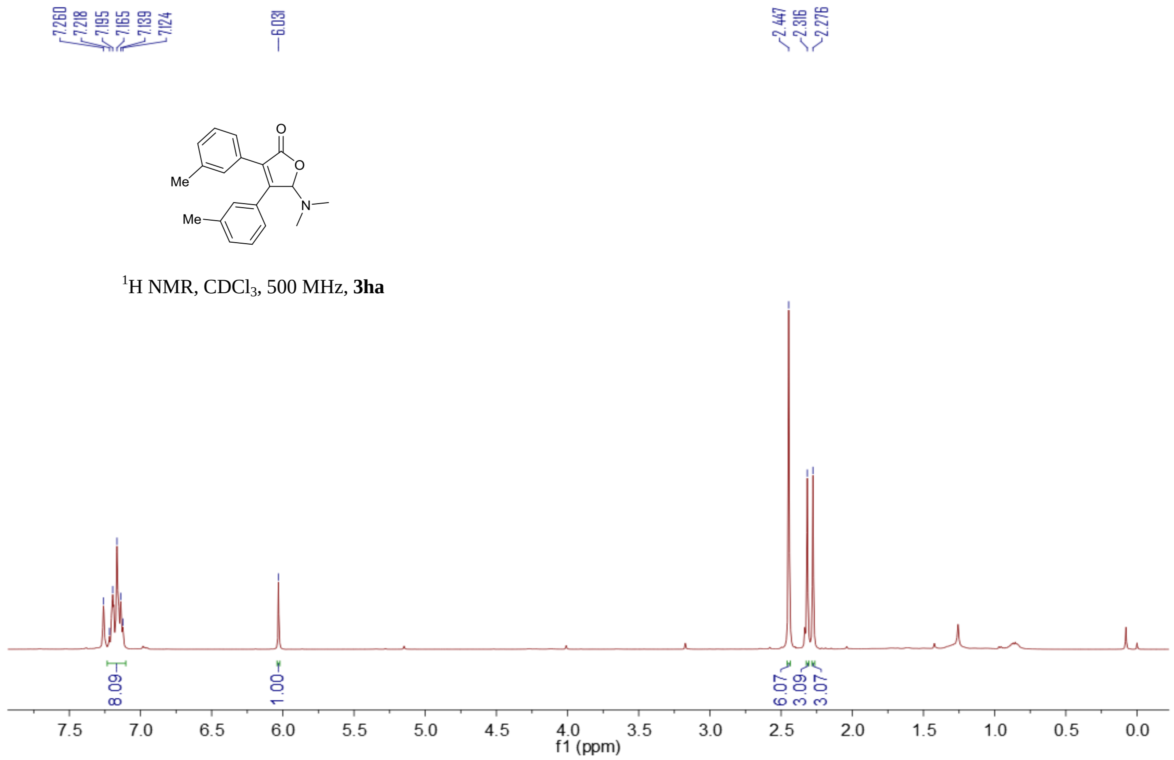

^{1}H NMR, $CDCl_3$, 500 MHz, **3ha**

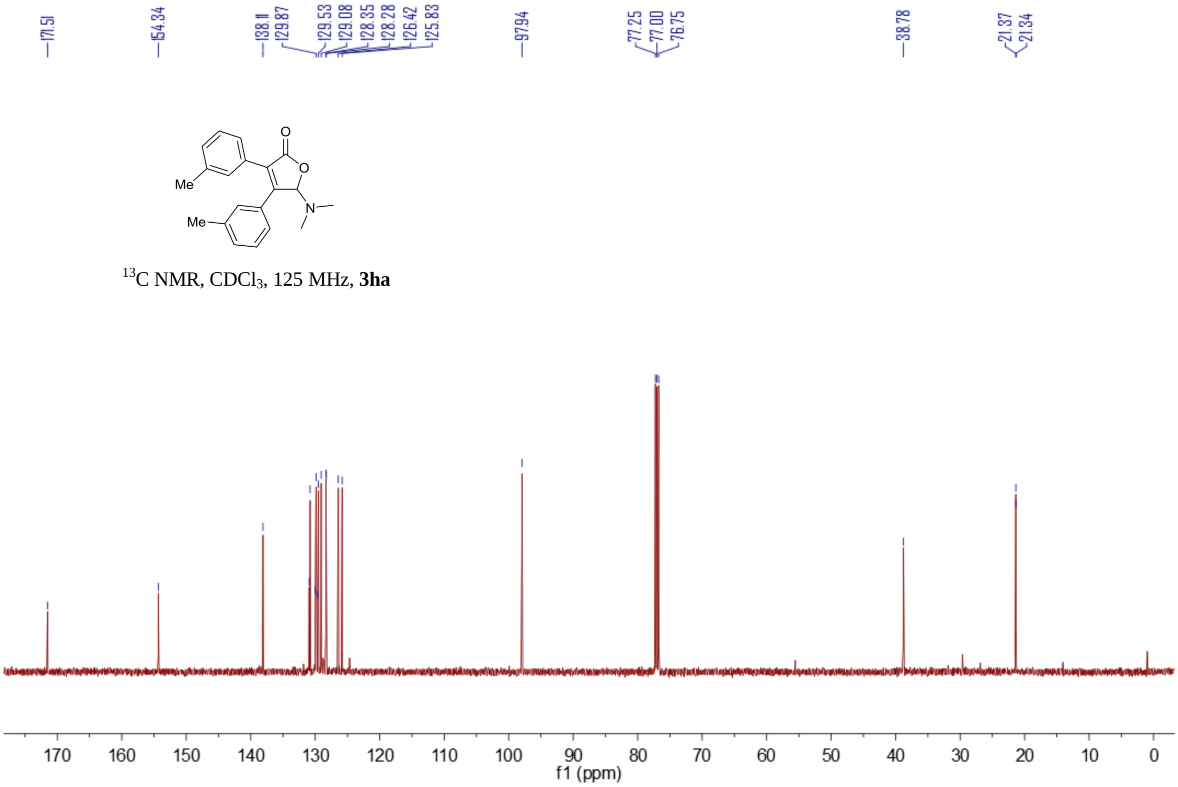

^{13}C NMR, $CDCl_3$, 125 MHz, **3ha**

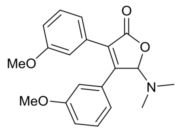

^{1}H NMR, $CDCl_3$, 500 MHz, **3ia**

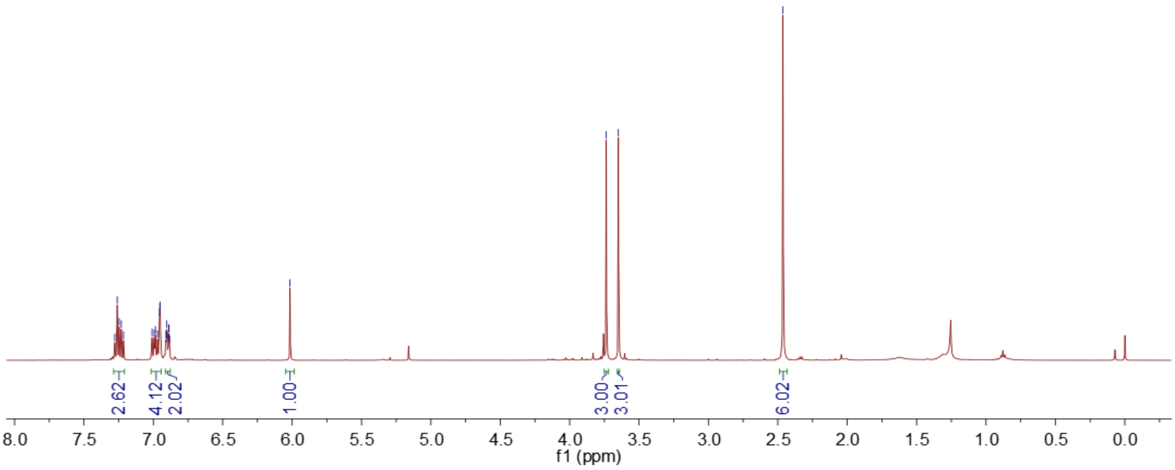

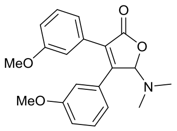

^{13}C NMR, $CDCl_3$, 125 MHz, **3ia**

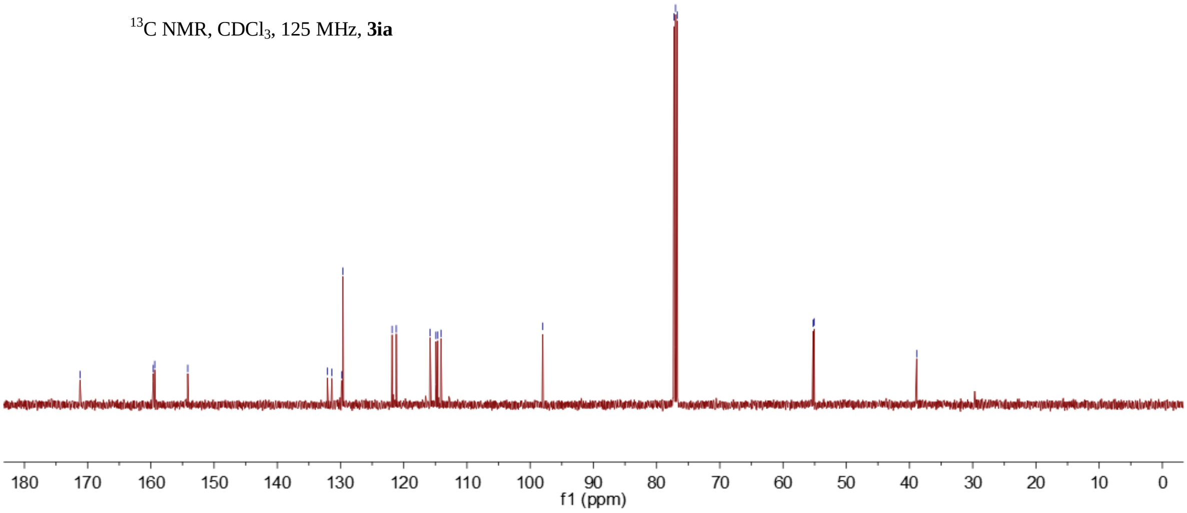

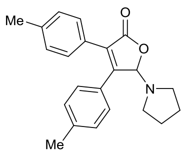

^{1}H NMR, $CDCl_3$, 500 MHz, **3bd**

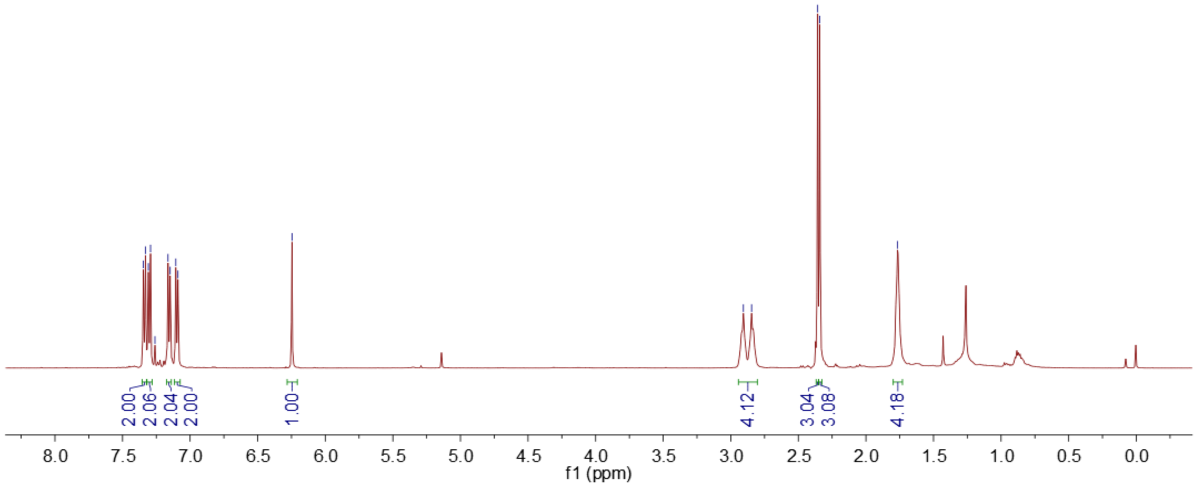

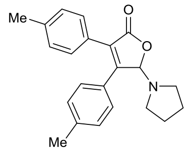

^{13}C NMR, $CDCl_3$, 125 MHz, **3bd**

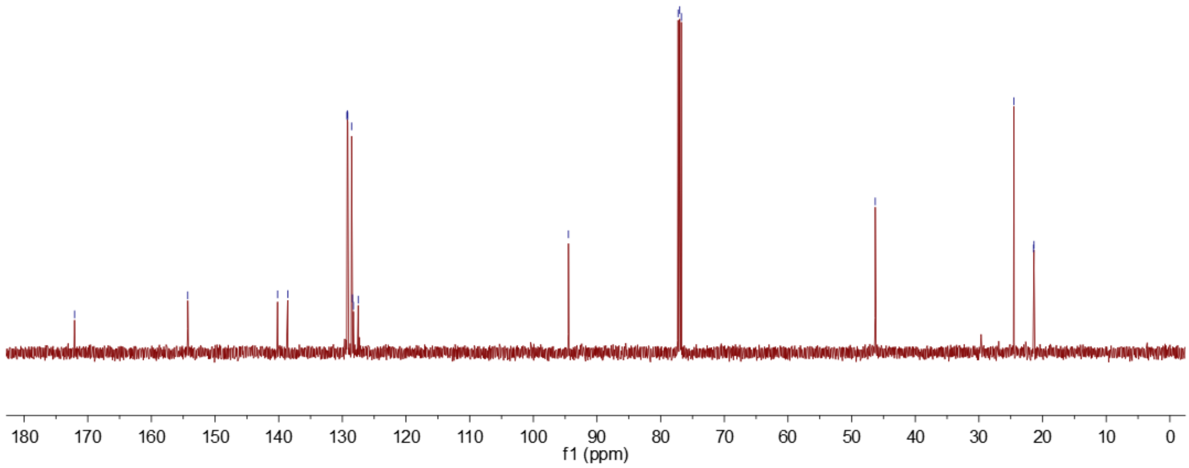

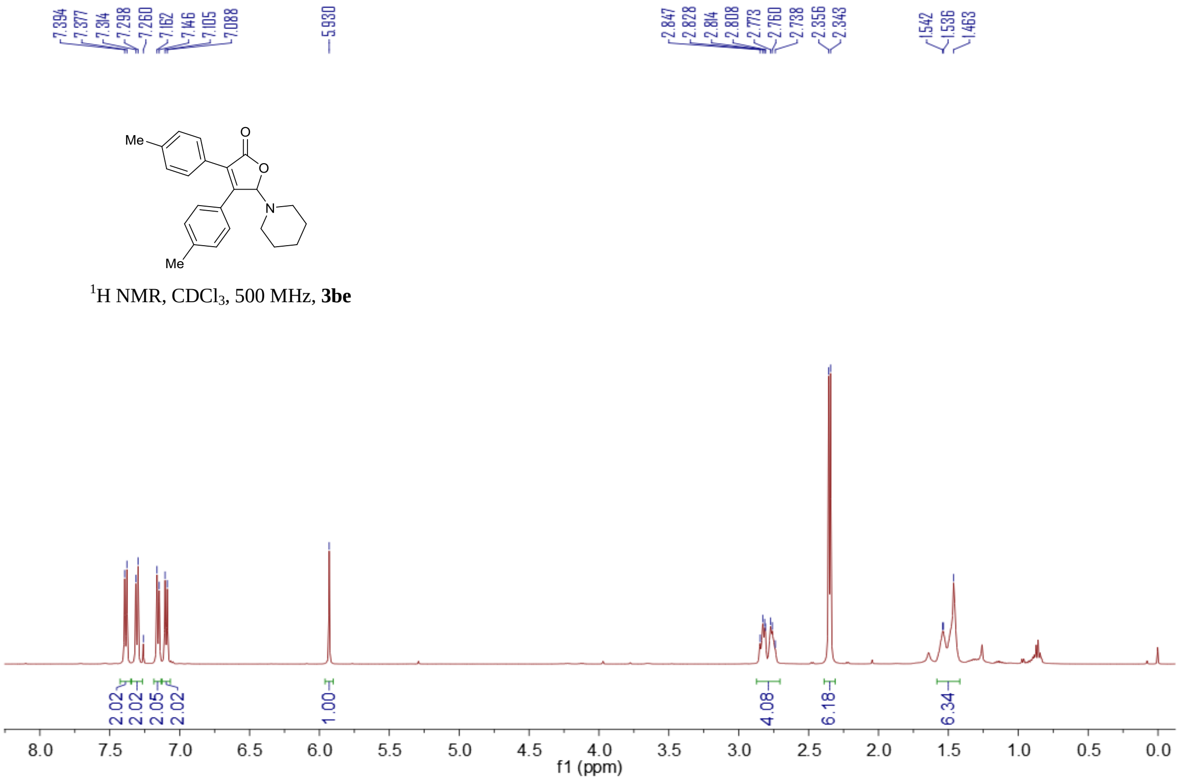

^{1}H NMR, $CDCl_3$, 500 MHz, **3be**

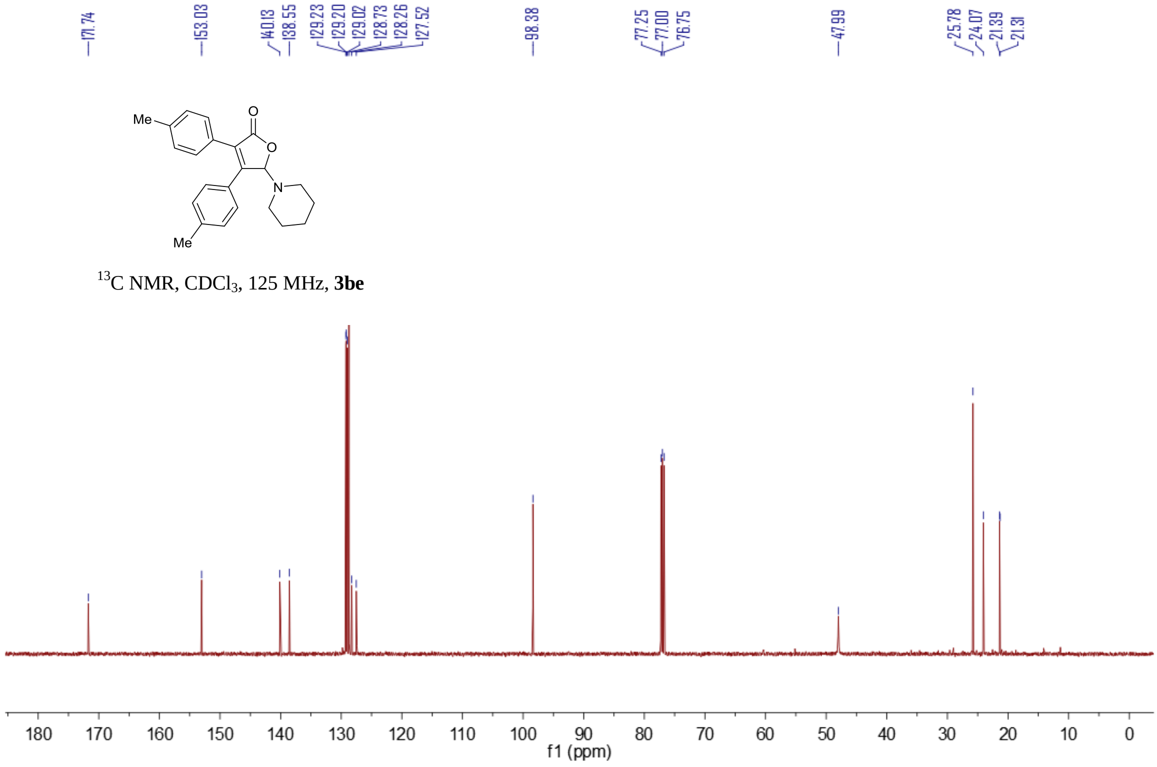

^{13}C NMR, $CDCl_3$, 125 MHz, **3be**

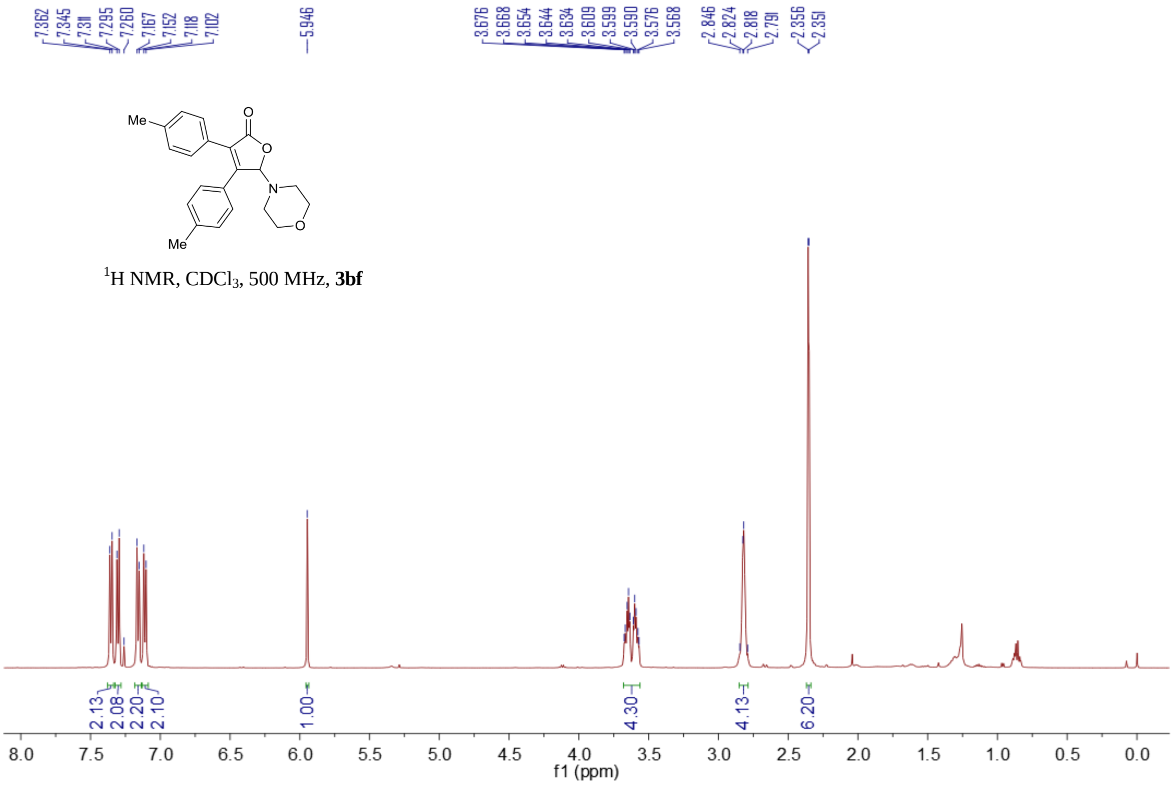

^{1}H NMR, $CDCl_3$, 500 MHz, **3bf**

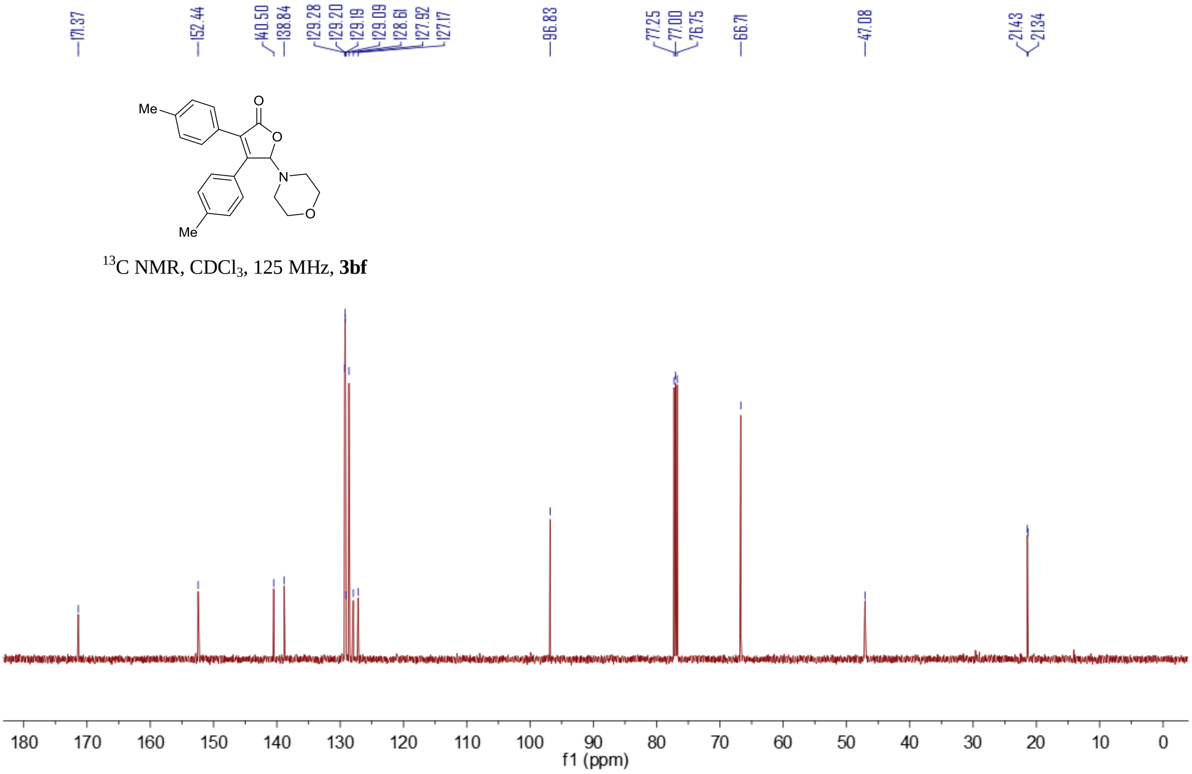

^{13}C NMR, $CDCl_3$, 125 MHz, **3bf**

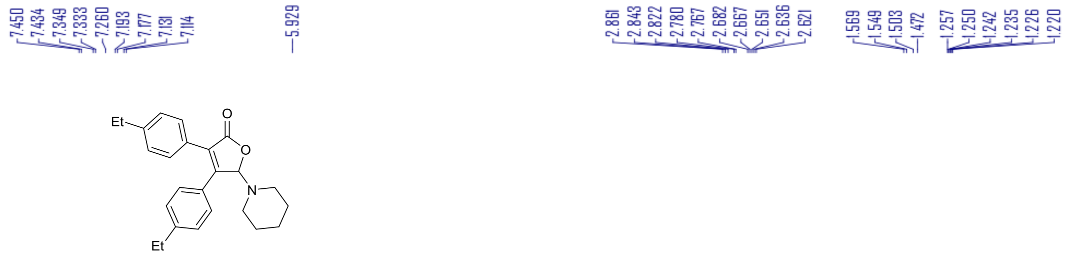

^{1}H NMR, $CDCl_3$, 500 MHz, **3ce**

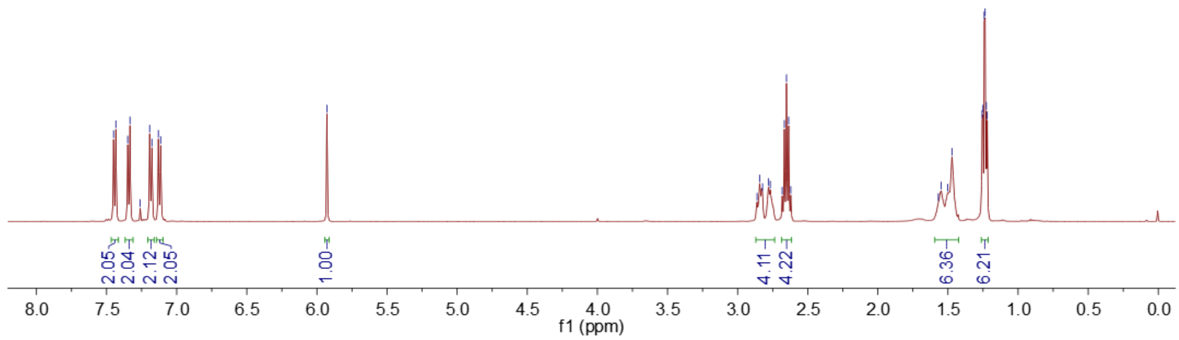

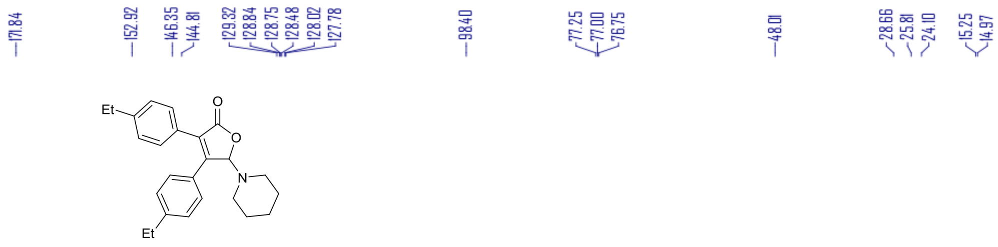

^{13}C NMR, $CDCl_3$, 125 MHz, **3ce**

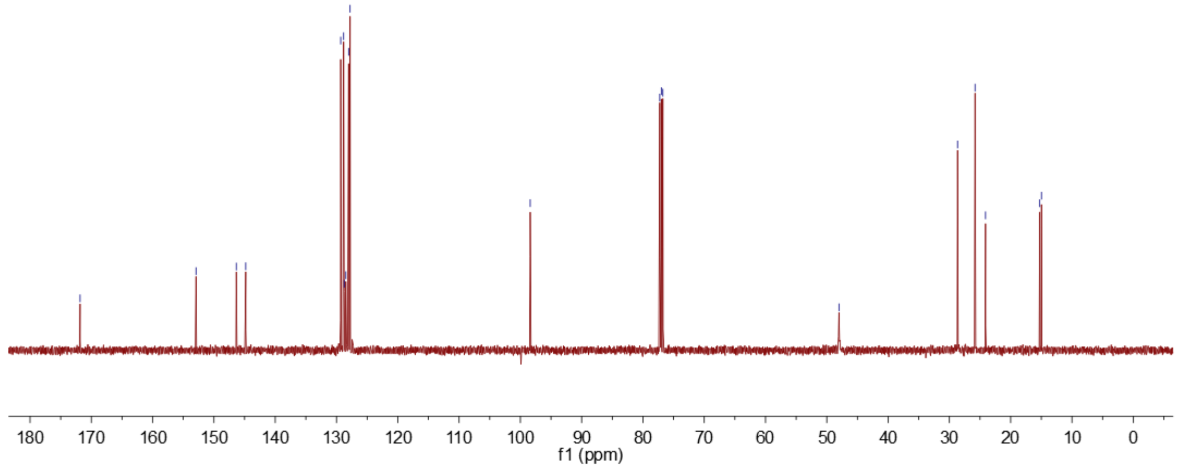

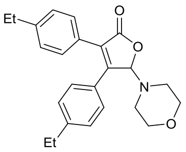

^{1}H NMR, $CDCl_3$, 500 MHz, **3cf**

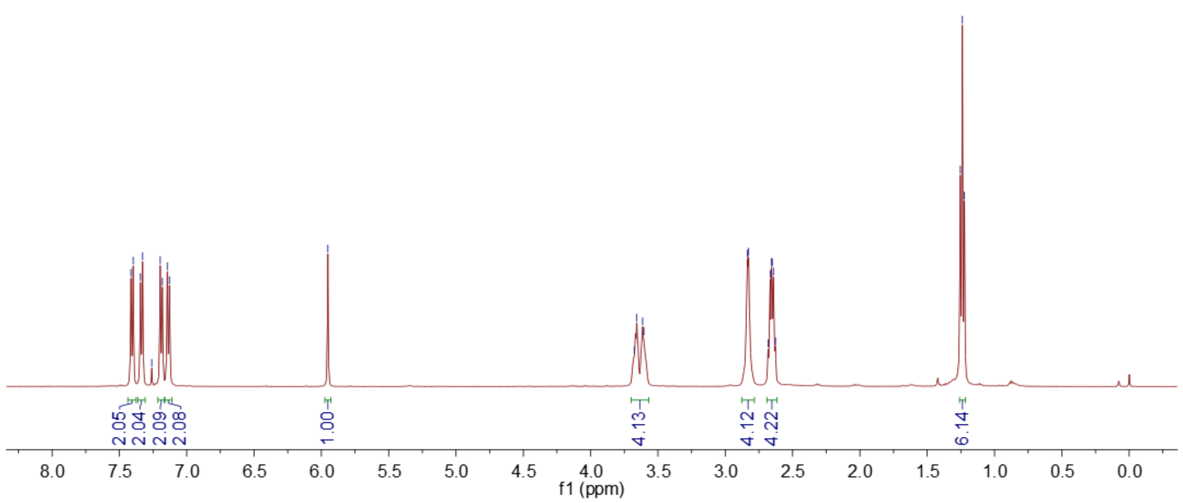

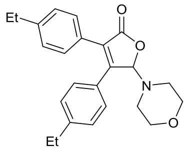

^{13}C NMR, $CDCl_3$, 125 MHz, **3cf**

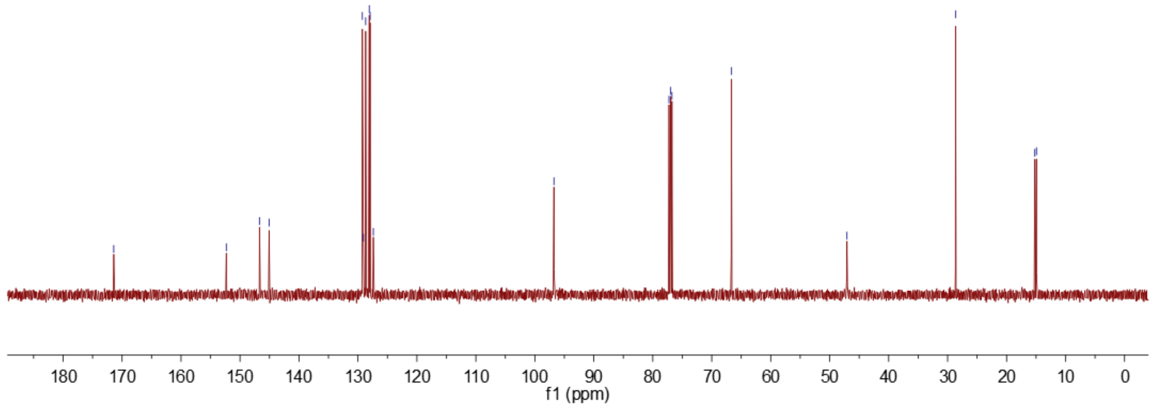

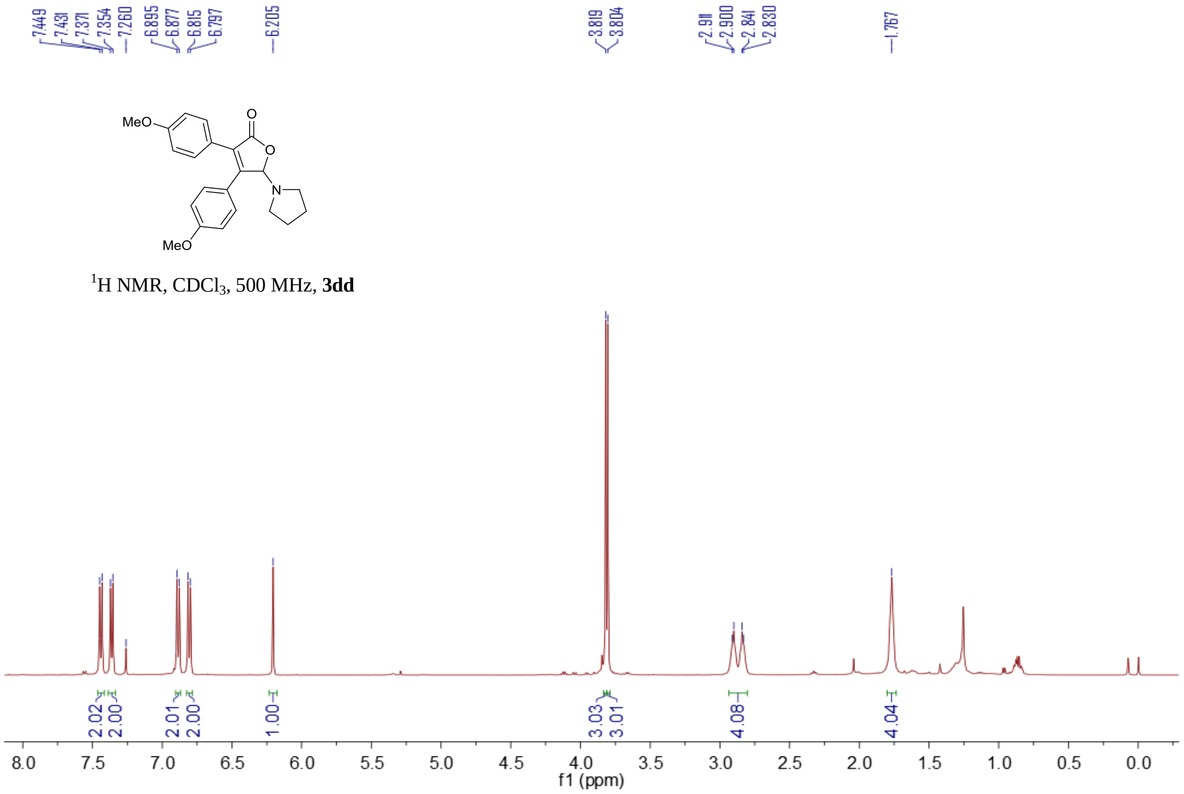

^{1}H NMR, CDCl$_3$, 500 MHz, **3dd**

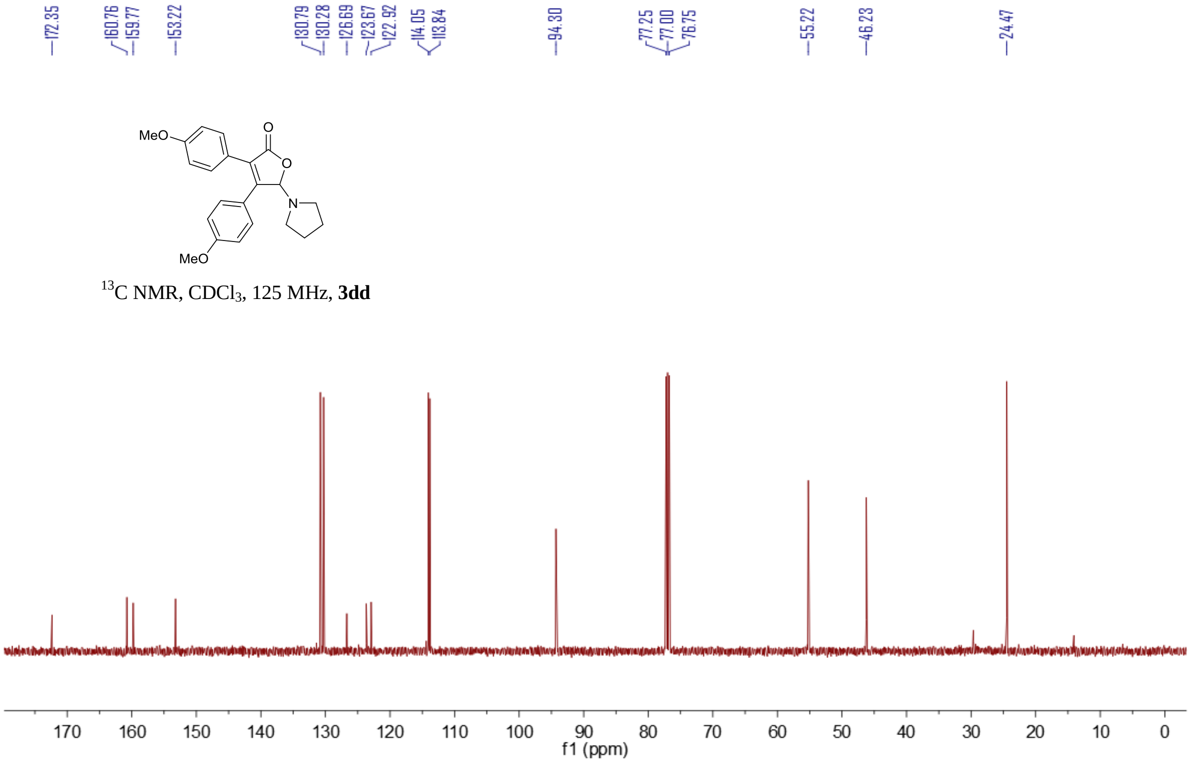

^{13}C NMR, CDCl$_3$, 125 MHz, **3dd**

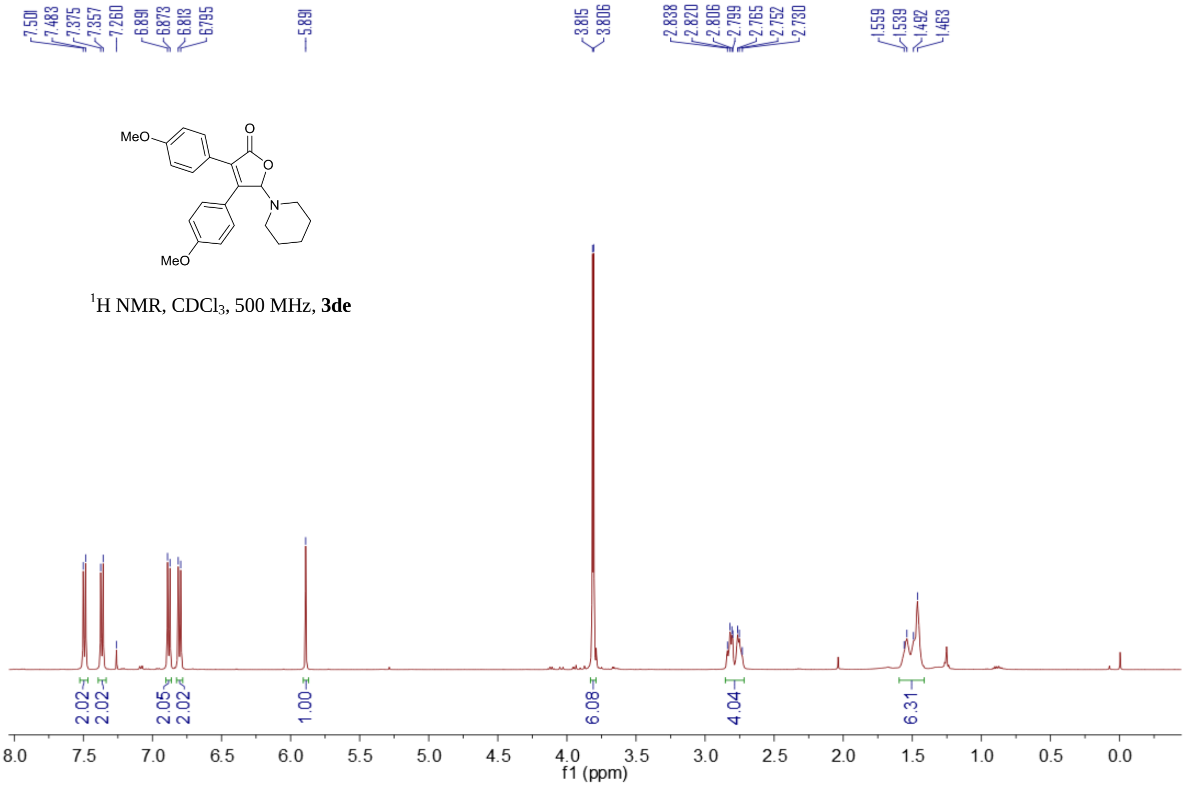

^{1}H NMR, $CDCl_3$, 500 MHz, **3de**

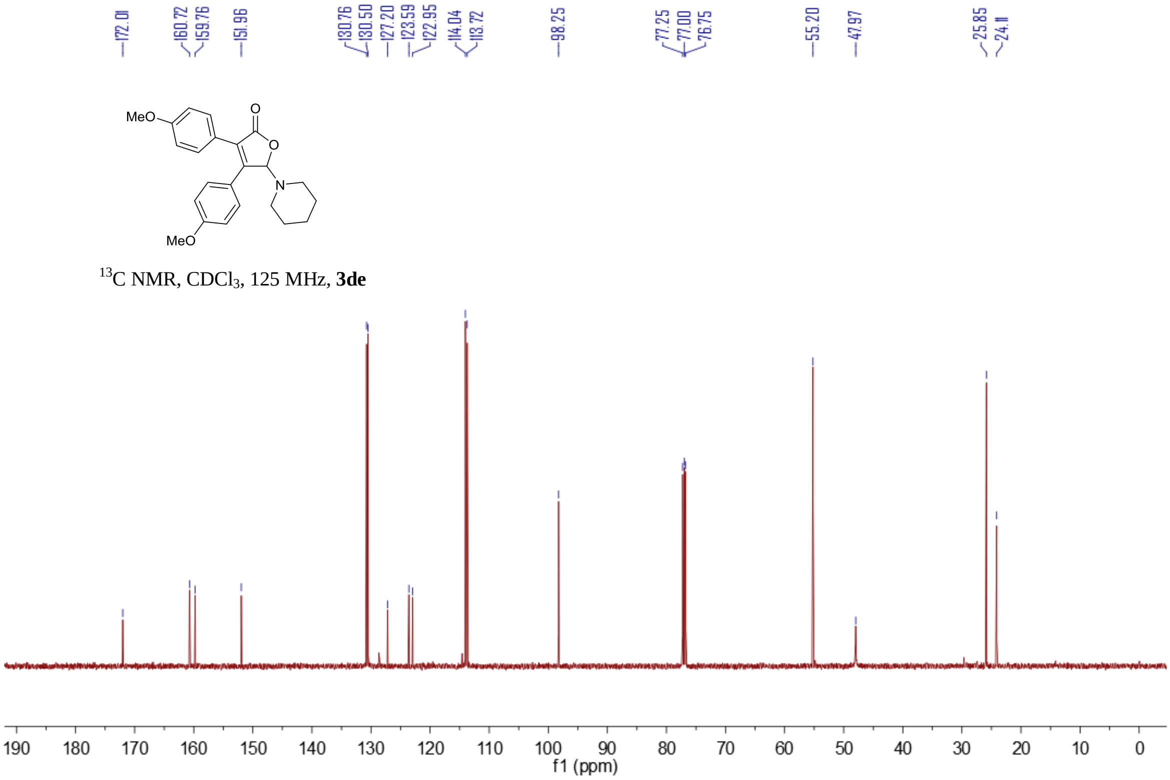

^{13}C NMR, $CDCl_3$, 125 MHz, **3de**

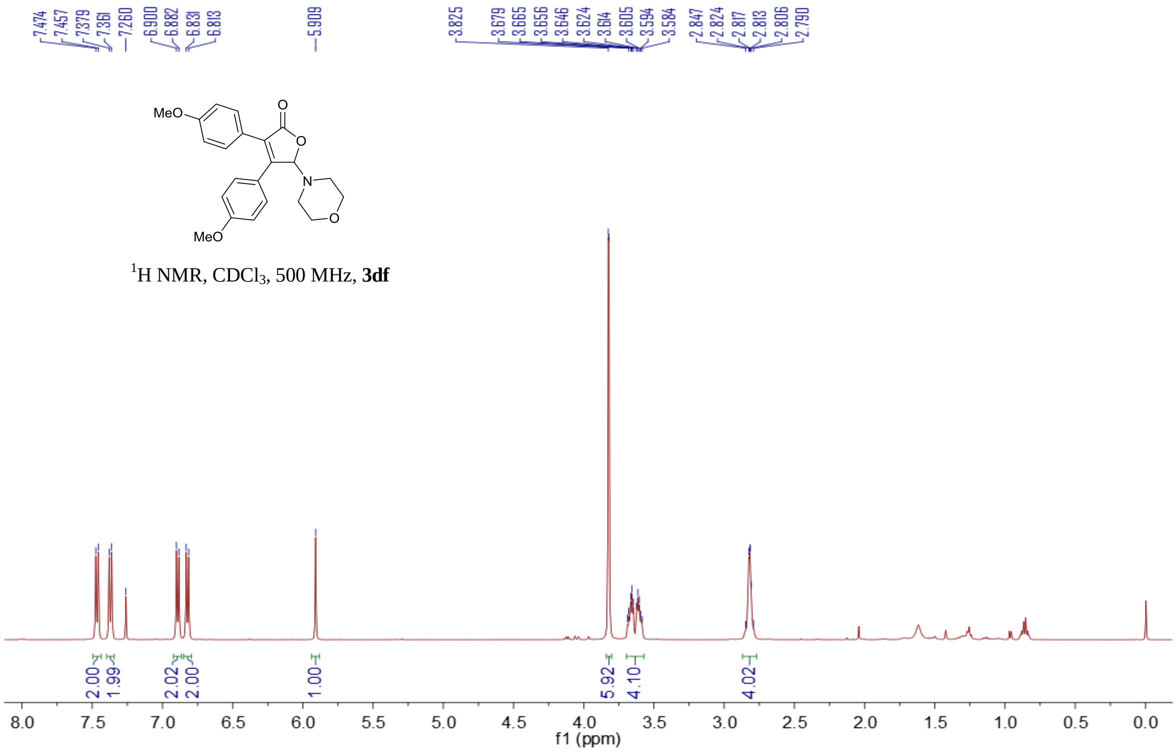

^{1}H NMR, $CDCl_3$, 500 MHz, **3df**

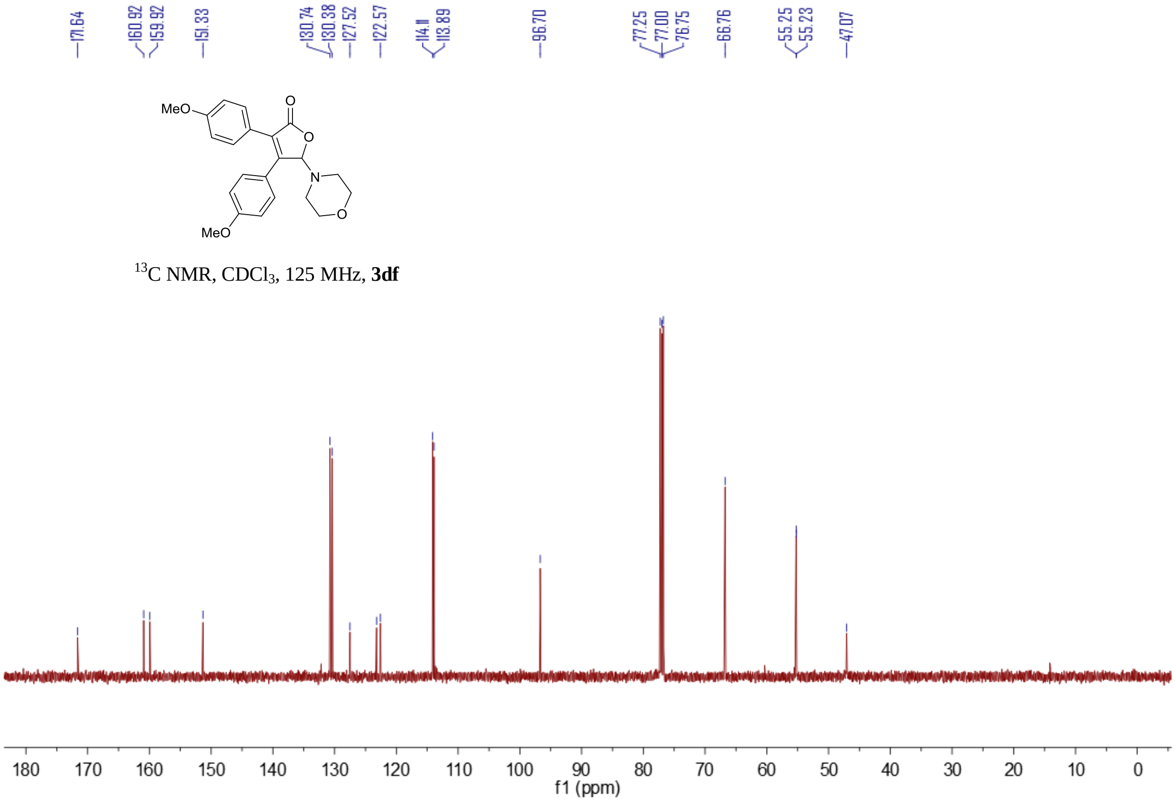

^{13}C NMR, $CDCl_3$, 125 MHz, **3df**

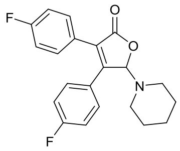

^{1}H NMR, $CDCl_3$, 500 MHz, **3ee**

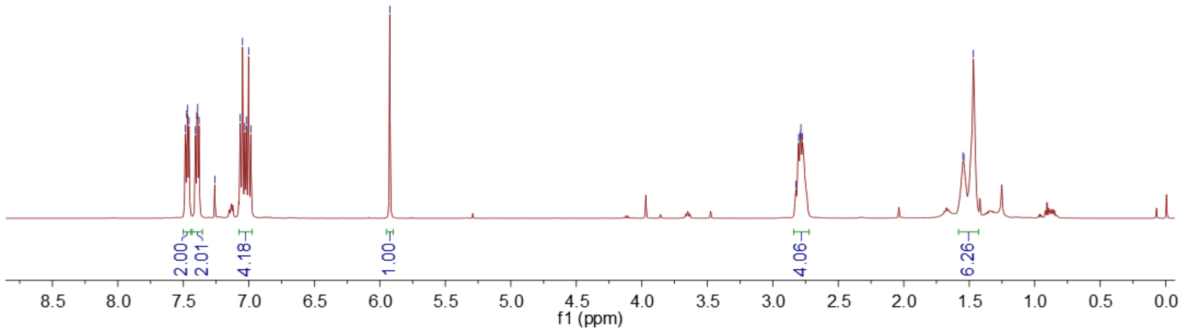

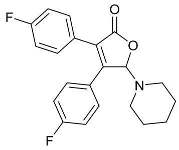

^{13}C NMR, $CDCl_3$, 125 MHz, **3ee**

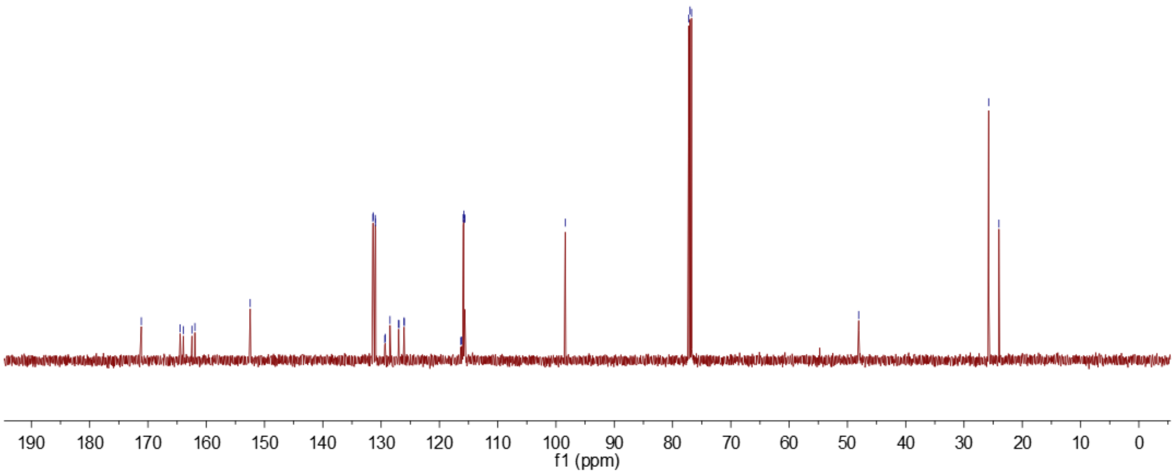

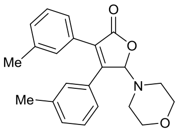

^{1}H NMR, $CDCl_3$, 500 MHz, **3hf**

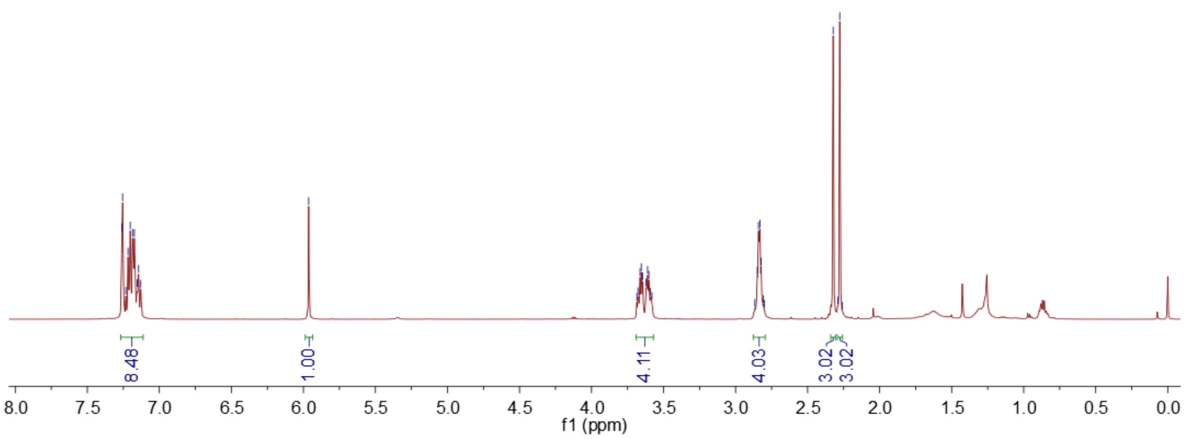

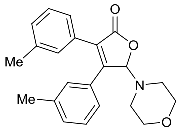

^{13}C NMR, $CDCl_3$, 125 MHz, **3hf**

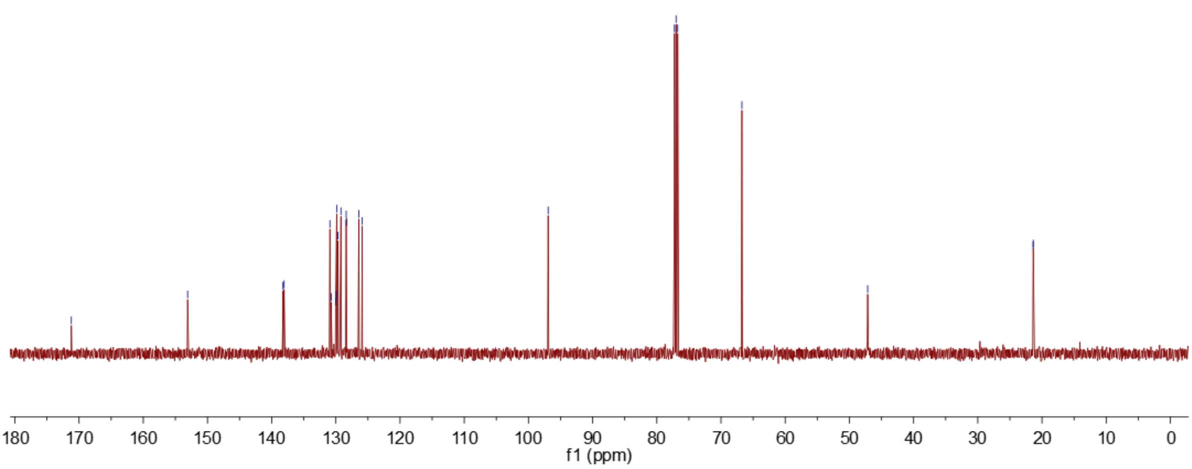

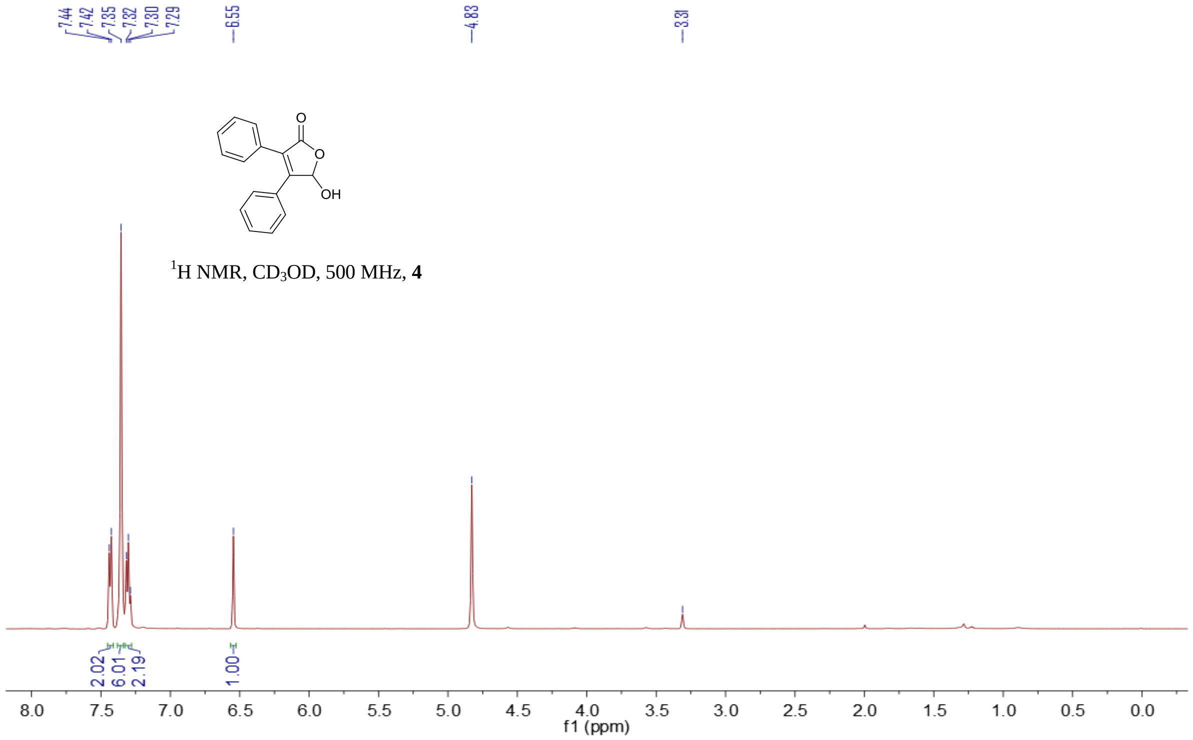

^{1}H NMR, CD_3OD, 500 MHz, **4**

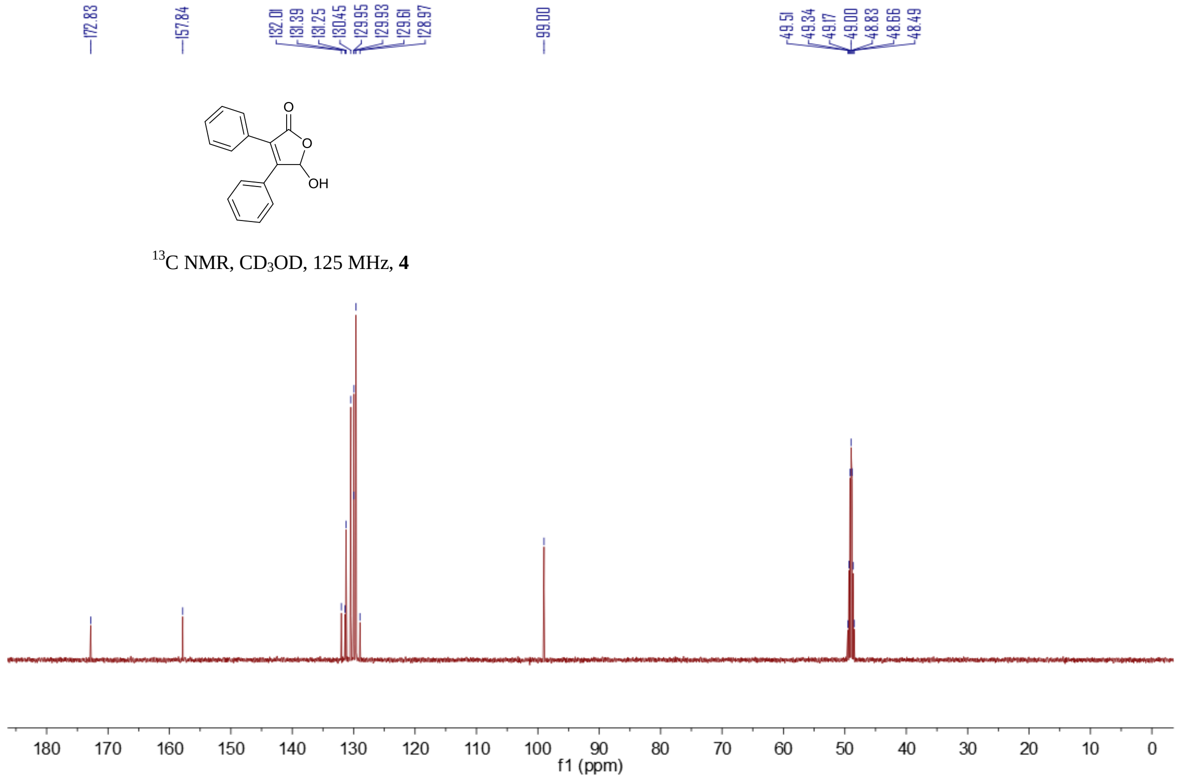

^{13}C NMR, CD_3OD, 125 MHz, **4**

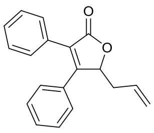

^{1}H NMR, $CDCl_3$, 500 MHz, **5**

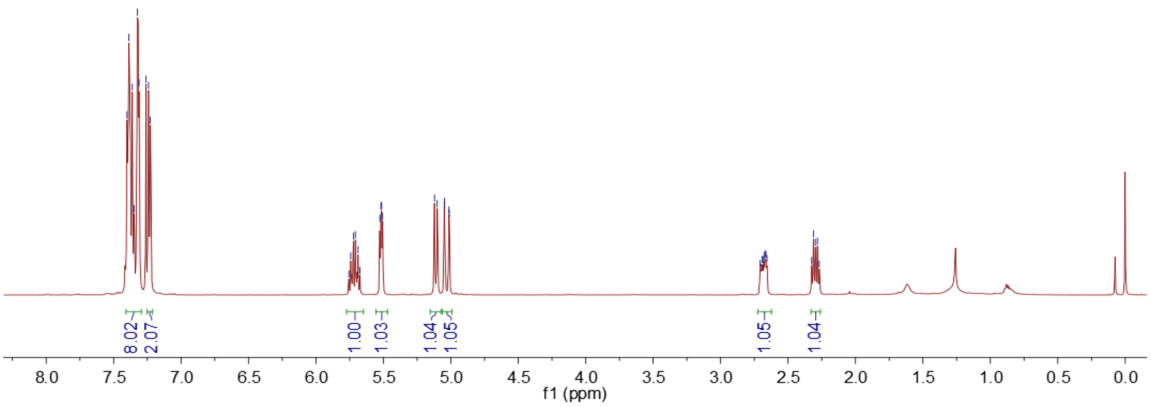

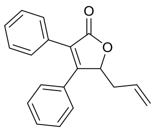

^{13}C NMR, $CDCl_3$, 125 MHz, **5**

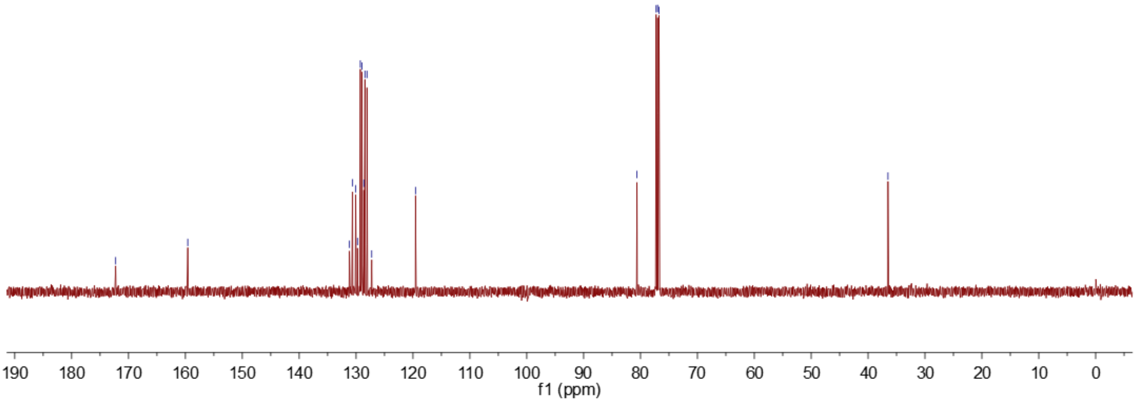

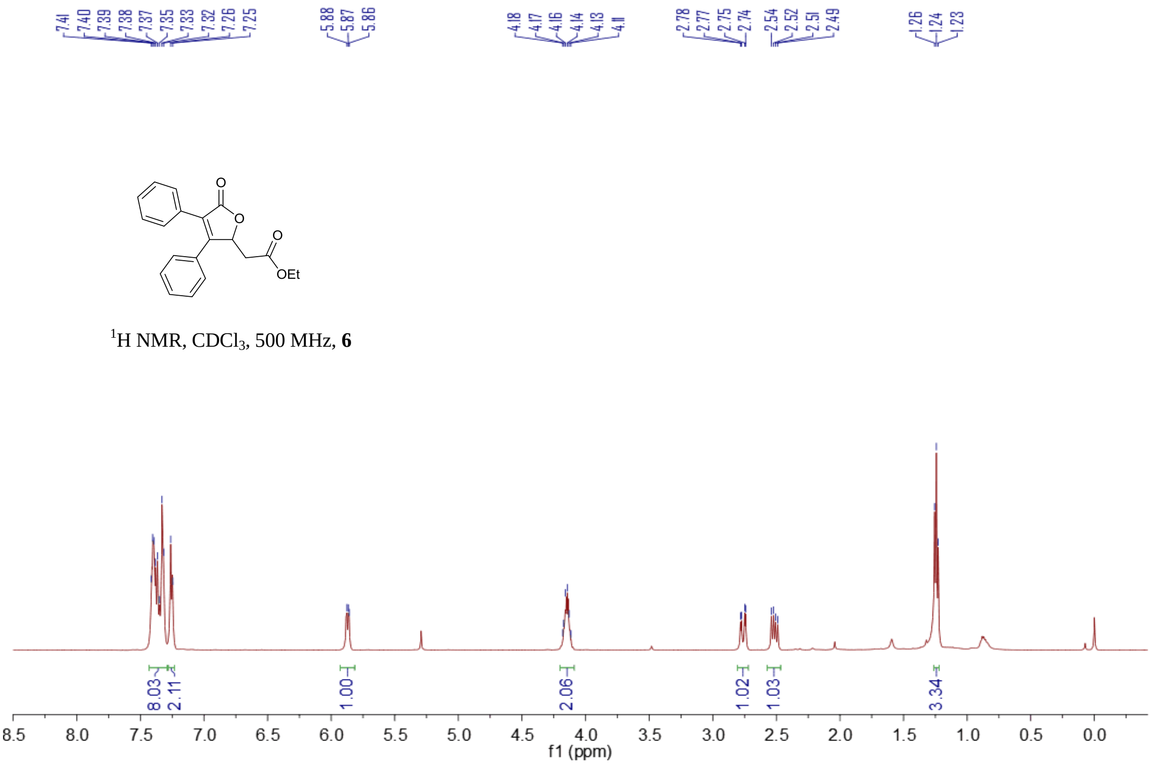

^{1}H NMR, $CDCl_3$, 500 MHz, **6**

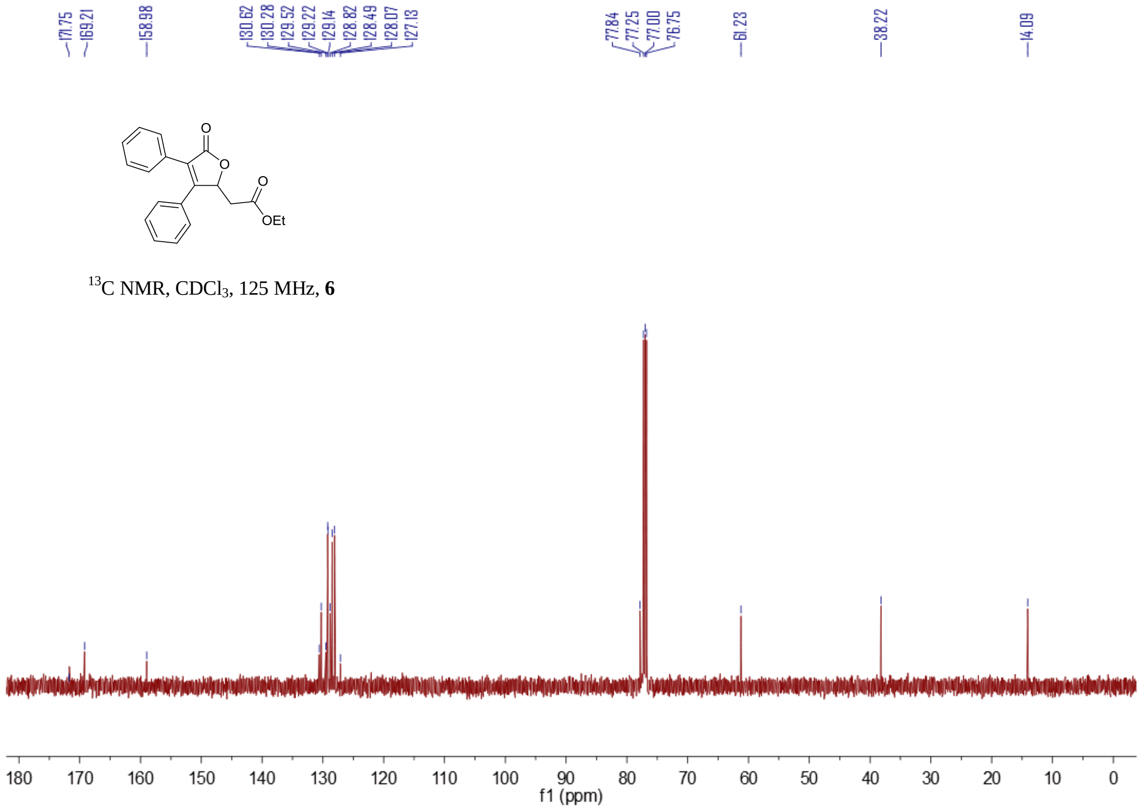

^{13}C NMR, $CDCl_3$, 125 MHz, **6**
